# Determining the quality and complexity of next-generation sequencing data without a reference genome

**DOI:** 10.1186/s13059-014-0555-3

**Published:** 2014-12-17

**Authors:** Seyed Yahya Anvar, Lusine Khachatryan, Martijn Vermaat, Michiel van Galen, Irina Pulyakhina, Yavuz Ariyurek, Ken Kraaijeveld, Johan T den Dunnen, Peter de Knijff, Peter AC ’t Hoen, Jeroen FJ Laros

**Affiliations:** Department of Human Genetics, Leiden University Medical Center, Leiden, The Netherlands; Leiden Genome Technology Center, Leiden University Medical Center, Leiden, The Netherlands; Department of Ecological Science, VU University Amsterdam, Amsterdam, The Netherlands; Department of Clinical Genetics, Leiden University Medical Center, Leiden, The Netherlands

## Abstract

We describe an open-source kPAL package that facilitates an alignment-free assessment of the quality and comparability of sequencing datasets by analyzing *k*-mer frequencies. We show that kPAL can detect technical artefacts such as high duplication rates, library chimeras, contamination and differences in library preparation protocols. kPAL also successfully captures the complexity and diversity of microbiomes and provides a powerful means to study changes in microbial communities. Together, these features make kPAL an attractive and broadly applicable tool to determine the quality and comparability of sequence libraries even in the absence of a reference sequence. kPAL is freely available at https://github.com/LUMC/kPAL.

**Electronic supplementary material:**

The online version of this article (doi:10.1186/s13059-014-0555-3) contains supplementary material, which is available to authorized users.

## Background

During the past decade, DNA sequencing technologies have undergone notable improvements with great impacts on molecular diagnostics and biomedical and biological research. Today, next-generation sequencing (NGS) technologies can provide insights into sequence and structural variations by achieving unprecedented genome and transcriptome coverage. Despite molecular and computational advances, the fast growing developments in library preparation, sequencing chemistry and experimental settings are of concern as they can diversify the complexity and quality of sequencing data [1-3]. To address data quality, most strategies rely on basic statistics of the raw data, such as the quality scores associated with base calling, the total number of reads and average GC content. Technical artefacts are usually only spotted after mapping of reads to the reference genome. However, such approaches are prone to alignment biases and the loss of potentially valuable information due to the predisposed and incomplete reference genome sequences [4-6]. These biases are considerably more problematic in studies of microbiomes as the species diversity can be immense [7], whereas the evaluation of data complexity and quality is limited to the analysis of species for which a reference genome sequence is available.

Analyzing the *k*-mer (DNA words of length *k*) frequency spectrum of the sequencing data provides a unique perspective on the complexity of the sequenced genomes, with more complex ones showing a greater diversity in unique sequences and repeated structures. Over- and under-represented *k*-mers have been associated with the presence of functional or structural elements (such as repetitive, mobile or regulatory elements), negative selection, or the hypermutability of CpGs [8-12]. Notably, the prevalence of functional elements and those caused by neutrally evolving DNA (including duplications, insertions, deletions and point mutations) is reflected in the modality (number of peaks) of the *k*-mer frequency spectrum [13,14]. The modality of the human genome is also subjected to its function as all coding regions, including the 5′ untranslated regions (UTRs), exhibit a unimodal k-mer spectrum, while the introns, 3′ UTRs and other intergenic regions have a multimodal distribution [13,14].

In recent years, *k*-mers have been used in a wide range of applications from the identification of regulatory elements to correction of sequencing errors, genome assembly, phylogeny analysis and the search for homologous regions [15-21]. It has also been shown that the characterization and comparative analysis of the *k*-mer spectrum can provide an unbiased view of genome size and structure, but it can also expose sequencing errors [22]. However, to our knowledge, most tools fail to accommodate for differences in library size and do not reliably expose problematic samples nor provide information on potential sources of variation in series of sequencing data. Here, we present a method, *k*-mer Profile Analysis Library (kPAL), for assessing the quality and complexity of sequencing data without requiring any prior information about the reference sequence or the genetic makeup of the sample. The proposed method uses the *distance* between *k*-mer frequencies to measure the level of dissimilarity within or between *k*-mer profiles. Since most distance measures are susceptible to differences in library size, we have implemented a series of functions that ensure a more reliable assessment of the level of dissimilarity between *k*-mer profiles. Based on the same principle, kPAL can identify problematic samples, as their level of similarity reduces in the absence of a significant difference between the genome of the sequenced samples. In this work, we apply kPAL to four types of NGS data: 665 RNA sequencing (RNA-Seq) samples [23,24], 49 whole genome sequencing (WGS) samples, 43 whole exome sequencing (WES) samples, and a series of microbiomes. We report the sources of technical and biological variation present in each set of NGS data, highlight a series of artefacts that were missed by standard NGS quality control (QC) tools, and demonstrate how the complexity of microbiomes is reflected in their *k*-mer profiles.

## Results and discussion

### Principles of kPAL

**Figure 1:**
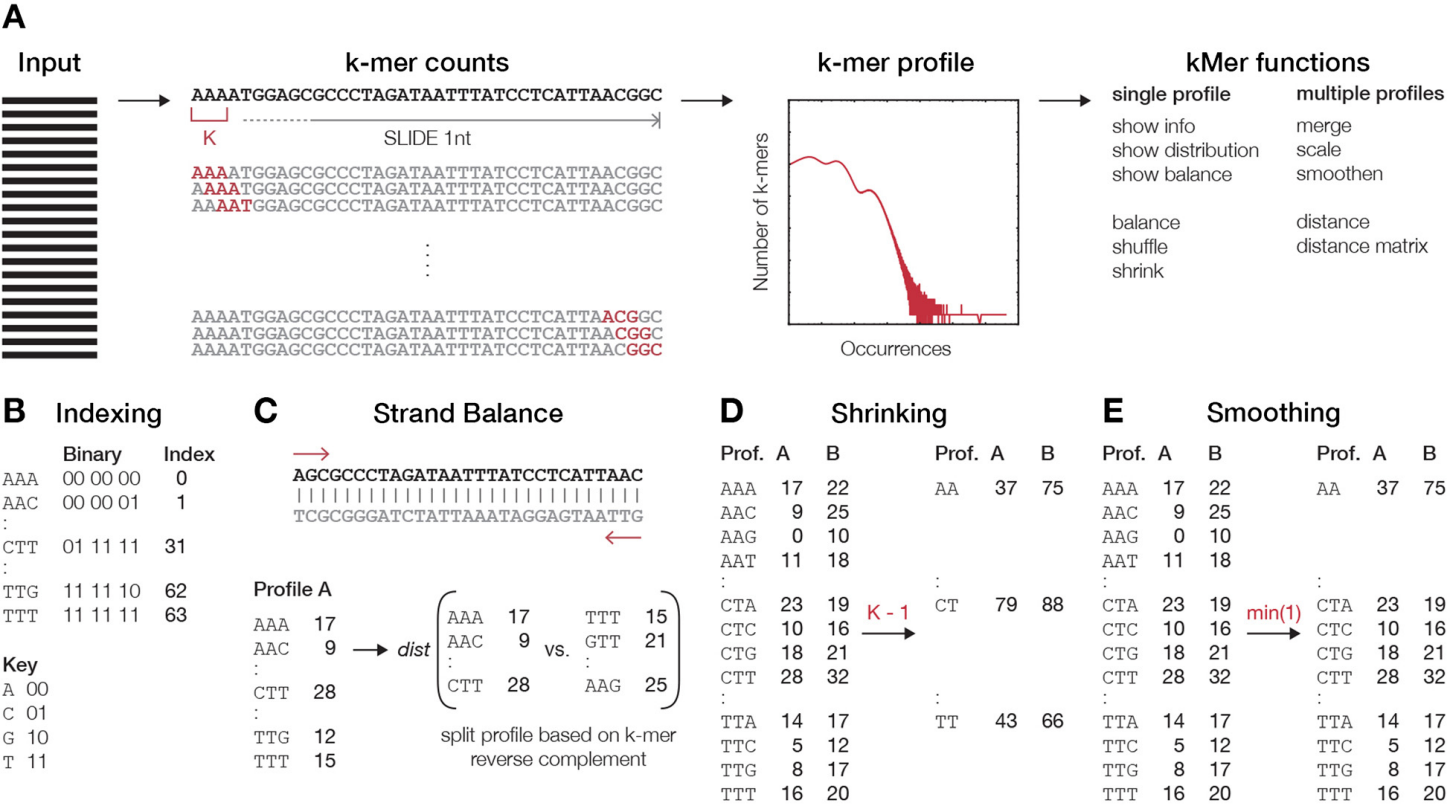
**Schematic overview of main kPAL principles. (A)** An overview of the procedure used by kPAL to assess the frequency of all *k*-mers within sequencing data. *k*-mers are identified and counted by a sliding window of size *k*. The *k*-mer spectrum can then be produced using the *k*-mer frequencies. The main functions of kPAL can be divided by their application to single or multiple profiles. For single *k*-mer profiles, general information about the number of nullomers, total number of counts, distribution of *k*-mer counts and balance between sequencing information from the plus and minus strands can be obtained with dedicated functions. If needed, profiles can be manipulated by the *balance*, *shuffle* and *shrink* functions. The balance function uses a sum of *k*-mers and their reverse complements to enforce balance between sequence information from the minus or plus strand. The shuffle function is designed to produce random *k*-mer profiles without changing the overall distribution of counts. **(B)** kPAL efficiently processes *k*-mers, as it encodes the sequences with a binary code using specific keys that can also facilitate a quick conversion to the reverse complement. Each nucleotide is represented by a binary code that is subsequently used to construct each *k*-mer. **(C)** The strand balance of a given *k*-mer profile is the overall distance measure between the frequency of the unique *k*-mer and its reverse complement. Thus, *k*-mer profiles are split into two sub-profiles that are reverse complements of each other and these are used to calculate the strand balance. **(D)** By design, kPAL can shrink *k*-mer profiles of size *k* to any smaller size. Counts from *k*-mers that share the first (*n* – 1) nucleotides are merged to collapse *k*-mer profiles to a size *k* – 1. **(E)** The smoothing function borrows the utility of shrinking and applies it locally to only *k*-mers that have lower counts than one defined by the user. Thus, for those affected, *k*-mer counts are merged and dropped to the size *k* – 1. The smoothing function accepts thresholds for the minimum, maximum or average counts of *k*-mers that share the first (*n* – 1) nucleotides but it also accepts user-defined functions. This process reiterates until the threshold condition is met. Prof., profile.

We developed an open-source package kPAL, which provides a series of tools (such as distance calculation, smoothing and balancing) to investigate the spectrum of *k*-mers observed in a given NGS dataset (Figure 1A and Additional file 1: Notes). The resulting *k*-mer profile holds valuable information on the complexity of the sequencing libraries and the sequenced genome(s). This is delineated in a graphical representation of the *k*-mer profiles, which plots the number of *k*-mers observed at each frequency. The complexity of genomic information is often reflected in the modality of this distribution, mainly due to repetitive and structural elements, and the context-specific composition of *k-*mers [10,13,14,25]. First, *k*-mers are processed using efficient binary codes that facilitate a rapid reverse complement conversion and access to specific *k*-mers (Figure 1B). Next, kPAL uses the distance between *k*-mer frequencies as a measure of dissimilarity between two *k*-mer profiles. In addition, calculating the correspondence between the frequencies of *k*-mers and their reverse complements aids in assessing the coverage balance between two strands of the sequenced library (Figure 1C). Generally, *k*-mer profiles can be shrunk to a smaller *k* size using the *shrink* function to enable access to smaller *k*-mer profiles without the need to reprocess the sequencing data (Figure 1D). However, it is important to note that large deviations from the original *k* size may obscure the true *k*-mer frequencies due to limited access to both ends of the sequencing reads (i.e., the last 12 nucleotides can be processed only once in a 12-mer profile whereas the same information is processed seven times in a 6-mer profile). To facilitate pairwise comparison of *k*-mer profiles and account for differences in library sizes, we have implemented complementary *scaling* and *smoothing* functions. Scaling *k*-mer frequencies to match the area under the curve of two profiles is a global normalization of the *k*-mer profiles. The smoothing function borrows the utility of shrinking and applies it locally to *k*-mers that have a frequency lower than a user-defined threshold, which results in local collapsing of those k-mers to a smaller size (i.e., *k* – 1) until the threshold condition is met (Figure 1E). For more information and a detailed explanation of kPAL features, see Additional file 1: Notes.

### Setting *k* size

To identify which *k* provides the best specificity for a mixed sample of bacteria, the *k*-mer profiles from three modelled metagenomes consisting of 30 bacterial genomes from the Firmicutes and Proteobacteria phyla (in 100:0, 50:50 and 0:100 ratios from each phylum) were compared to ten randomly shuffled sequences (without changing the overall nucleotide composition). The optimal value for *k* is the one that best separates metagenomes from randomly permuted sets. The overall distance between *k*-mer profiles of the metagenomes and the corresponding randomly permuted sets starts to level off once *k* exceeds 10 (Additional file 1: Figure S1). A low amount of variation in distance between the *k*-mer profiles of metagenomes and their permuted sets indicates that the distance measure is generally robust and only changes according to *k*. Interestingly, the optimal separation coincides with the *k* for which the complete unimodal spectrum of frequencies (from those that are too rare to those that are highly recurrent) is observed (Additional file 1: Figure S2A,B,C).

The human reference genome has a high complexity (described in Additional file 1: Notes), based on the multimodality of the *k*-mer profiles, which ranges from 9 to 15 (Additional file 1: Figure S3A). In humans, *k* = 11 is the smallest value for which unique *k*-mers and nullomers (absent *k*-mers) are observed while genomic spectra for *k* ≥ 13 start to lose their multimodality as they become too unique. Thus, *k* = 12 was used to give a relatively balanced number of nullomers, and unique and frequent *k*-mers. This allows for the identification of potential artefacts (mainly reflected by rare *k*-mers) as well as biological and contextual variations. Interestingly, the level of complexity varies between different types of genomic information (WGS, WES and RNA-Seq; see Additional file 1: Figure S3B). In contrast to genomic sequences, the coding part of the human genome exhibits a unimodal profile, as shown before [13,14]. The minor differences between the *k*-mer profiles of the exome and the transcriptome reference sequences are due to the number of shared coding regions between different transcript variants of the same gene. The transcriptome reference sequences generally exhibit higher counts for observed *k*-mers and lower numbers of nullomers introduced by exon–exon junctions. Moreover, the *k*-mer spectrum derived from sequencing data is in concordance with that of the reference (Additional file 1: Figure S3C). The minor deviations from the unimodality of the exome and transcriptome data are mainly due to the capture performance (off-target reads introduce low-count *k*-mers that represent intronic and intergenic regions) and differences in the abundance of expressed mRNA.

In addition to the complexity of the genomic information, the sequencing depth contributes to the modality and the resolution of the *k*-mer spectrum derived from individual datasets. In RNA-Seq, we observed that the number of 12-nullomers correlates with the total number of reads per dataset (*R* = −0.80; see Additional file 1: Figure S4A,B). The variation in the total read counts per sample is partly due to study design, as sequencing was performed in seven different laboratories [24]. Thus, the total number of 12-nullomers also varies between samples from different laboratories (Additional file 1: Figure S4C). It is crucial to account for bias introduced by poor and variable coverage, as it may obscure the identification of factors that determine the complexity of the *k-*mer spectrum. One obvious solution would be to opt for lower *k* sizes (i.e., *k* = 9) at the expense of specificity. However, we propose the dynamic *smoothing* function, which is resilient towards coverage bias and does not sacrifice the specificity of the *k*-mer spectrum by choosing a smaller *k* (Additional file 1: Notes). This function only shrinks the *k*-mer profile locally when the counts do not pass predefined conditions (i.e., they fall below an acceptable threshold for *k*-mer frequencies). In the next section, we show how kPAL can be used to assess the quality of different types of sequencing data without relying on the availability of a well-characterized reference genome.

### Evaluating data quality without a reference

**Figure 2:**
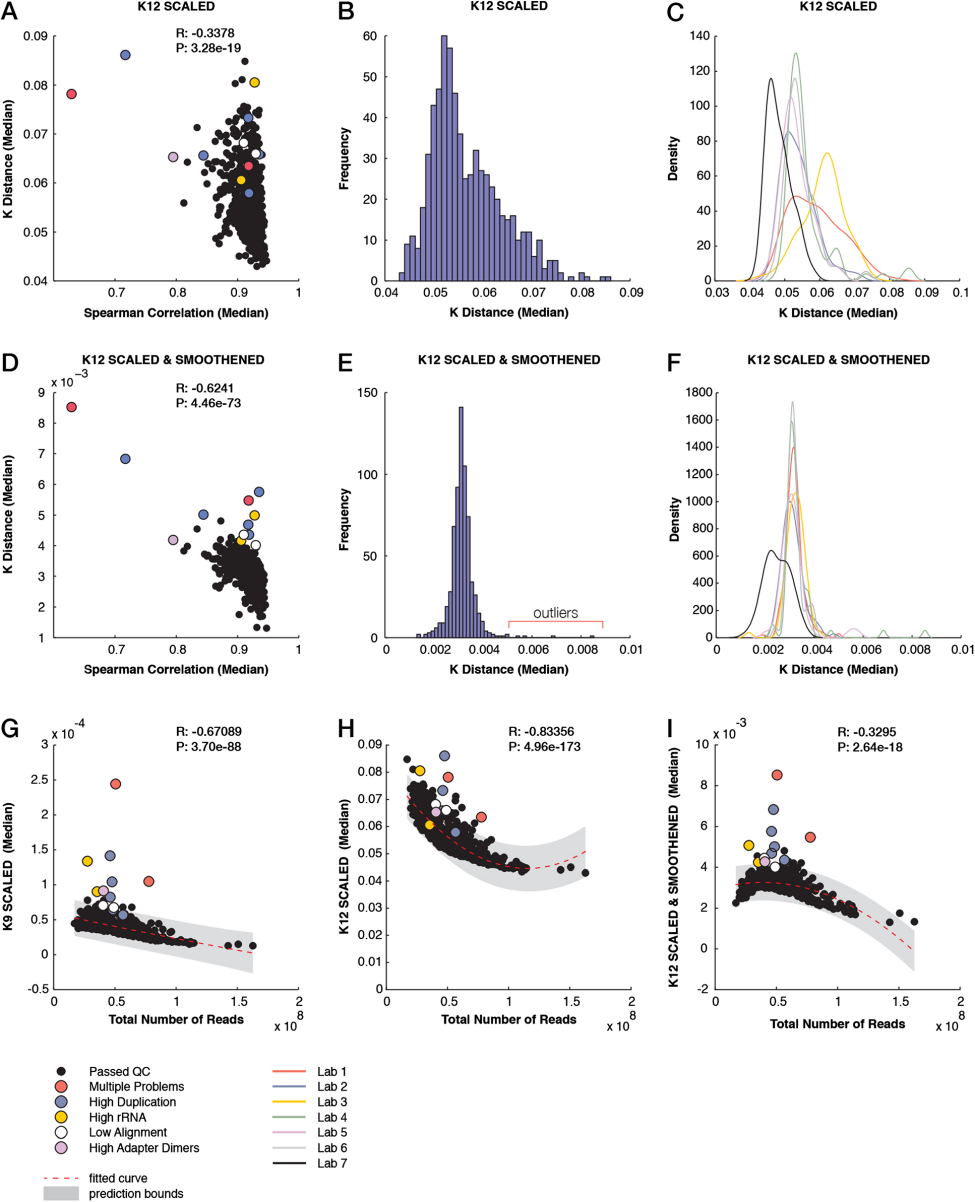
**Evaluating data quality for mRNA sequencing samples across different laboratories. (A)** Scatter plot showing for each sample the median pairwise Spearman correlation for exon quantification and the median *k*-mer distance measures (*K* distance) after scaling. Problematic samples are highlighted in different colors. **(B)** Histogram of median *K* distance (scaled) for each individual sample. **(C)** Distribution of median *K* distance (scaled) for each sequencing laboratory (indicated by different colors). **(D)** Scatter plot of median pairwise Spearman correlation between exon quantification and *K* distance (smoothed and scaled). **(E)** Histogram of median *K* distance (smoothed and scaled) for each individual sample. **(F)** Distribution of median *K* distance (smoothed and scaled) for each sequencing laboratory (indicated by different colors). **(G)** Scatter plot of the total number of reads per sample versus the *K* distance of 9-mers (scaled). The poly2 fitted line and the 95% confidence intervals are indicated. **(H)** Scatter plot of the total number of reads per sample versus the *K* distance of 12-mers (scaled). **(I)** Scatter plot of the total number of reads per sample versus the *K* distance of 12-mers (smoothed and scaled). Lab, laboratory; QC, quality control.

Recently, we showed that performing a pairwise comparison of 9-mer (K9) profiles, without alignment to the reference sequence, can expose quality issues in RNA-Seq data [24]. The median of all pairwise distances for each sample correlated (*R* = −0.63) with the correlation measures obtained after alignment and quantification of exon expression levels, which are post-alignment measures often used for QC. Notably, some of the problematic samples (due to a high duplication rate and/or high rRNA content) could only be identified by an analysis of their *k*-mer profiles. However, kPAL scores could not separate all problematic samples. Thus, we performed these analyses for larger values of *k* to increase the specificity and investigate whether smoothing can remove biases introduced by variable sequencing depth between samples. For 12-mer (K12) profiles, the distance measures calculated after scaling only showed a much weaker correlation (*R* = −0.34) with the correlation measures obtained from the exon quantification of samples (Figure 2A). They also displayed a broad distribution with no apparent clustering of known outliers (Figure 2B). We also observed a variation between samples based on the laboratory in which the sequencing was performed, mainly reflecting the library size differences (Figure 2C and Additional file 1: Figure S5A). After smoothing the *k*-mer profiles, the *k*-mer pairwise distances were in good concordance (*R* = −0.62) with the correlation measures of the exon quantifications obtained after alignment (Figure 2D). Smoothed K12 profiles exhibited a narrow distribution, having known problematic samples as only outliers (Figure 2E). Importantly, the variation between laboratories was significantly reduced as the dynamic smoothing function can accommodate differences in library size (Figure 2F and Additional file 1: Figure S5B). These median pairwise distances were far less sensitive to differences in the total read counts per sample than distances obtained from scaled 9-mer and 12-mer profiles (*R* = −0.33, −0.67 and −0.83, respectively; Figure 2G,H,I). Moreover, the number of known problematic samples that fall outside the 95% prediction bounds is improved to 11 (out of 12) in smoothed K12 distances compared to that of K9 and K12 (eight and five, respectively). The sample NA18861.4 has by far the highest distance to other samples in both K9 and smoothed K12 analyses (Figure 2G,I). We have previously reported that this sample has a significant genomic DNA contamination since only 4% of reads mapped to exons [24]. This contamination can affect the complexity of the sequenced library as many reads represent the non-coding and repetitive regions of the genome. Whereas samples that passed the QC measures exhibited *k*-mer spectra that reflected the expected modality of the transcriptome (Additional file 1: Figure S6A), the distribution of *k*-mer frequencies in NA18861.4 clearly mimicked that of the full human reference genome (Additional file 1: Figure S6B).

**Figure 3:**
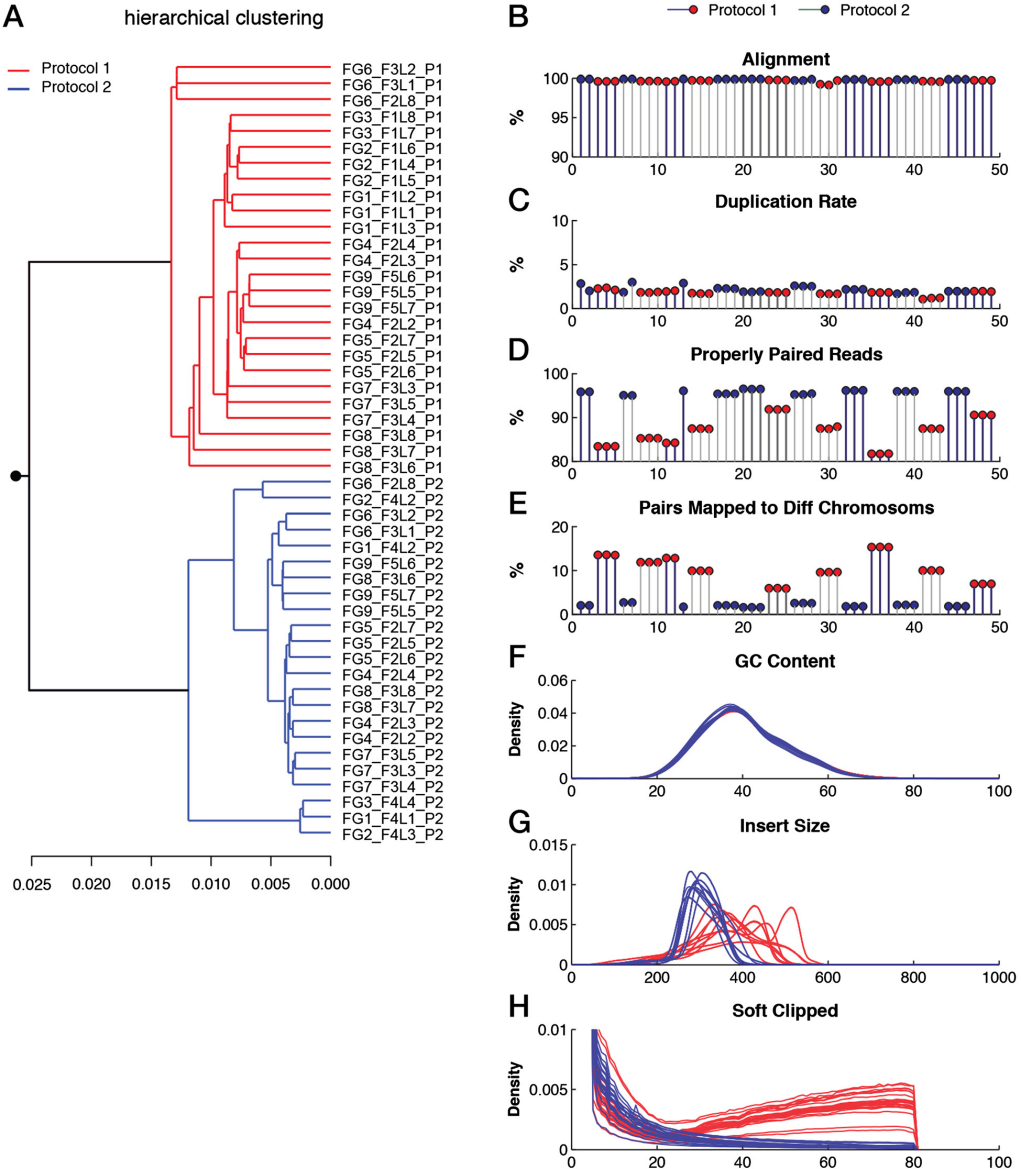
**Data quality and the influence of library preparation protocol in whole genome sequencing data. (A)** Hierarchical clustering of pairwise *k*-mer distance measures across WGS samples. Samples prepared using different protocols are indicated in different colors. **(B)** Percentage of aligned reads per sample. Black and grey bars separate samples from different individuals. Red and blue circles indicate the choice of library preparation protocol. **(C)** Percentage of duplicated reads. **(D)** Percentage of properly paired reads. **(E)** Percentage of paired reads that map to different chromosomes. **(F)** Distribution of average GC content per read. Samples prepared using different protocols are colored accordingly. **(G)** Distribution of estimated insert size. **(H)** Distribution of the number of base pairs that are soft clipped from reads during the alignment. Diff, different; WGS, whole genome sequencing.

We also addressed quality issues in WGS data. In our set of 49 WGS samples from nine individuals, pairwise distances between smoothed 12-mers clustered samples into two main groups that represent the choice of the library preparation protocol (Figure 3A). Within the cluster representing the first protocol, most datasets were further clustered on the individuals from whom the samples were obtained. Importantly, all datasets passed all the quality measures in the commonly used QC pipeline for NGS data, FastQC [26]. The alignment (99.7%), duplication rates (2.0%) and the overall GC content did not differ significantly between datasets (Figure 3B,C,F). However, datasets differed in the percentage of properly paired reads (86.7% and 95.8%) and pairs mapping to different chromosomes (10.6% and 2.1% for protocol 1 and protocol 2, respectively) based on the choice of library preparation protocol (Figure 3D,E). Pairs that mapped to different chromosomes did not cluster at specific loci but were distributed across the entire genome (Additional file 1: Figure S7). Moreover, the sequencing reads from the first protocol exhibited a bimodal and broader insert size distribution (Figure 3G and Additional file 1: Figure S8B). The enrichment of pairs that map to different chromosomes and the widening of the insert size distribution could indicate the presence of library chimeras (sequences derived from two or more different fragments). The number of soft clipping events (unmatched region of a partially aligned read, up to 80 base pairs long) during the alignment confirms the enrichment of library chimeras in samples that were prepared using the first protocol (Figure 3H). We ruled out the influence of aligner as the results obtained from three different aligners (Stampy, BWA and Bowtie2) were in concordance (Additional file 1: Figure S9A,B). Library chimeras and erroneous bases can potentially introduce artificial *k*-mers and therefore enrich for rare features in the *k*-mer spectrum. This is supported by the *k*-mer profiles of the samples from the two library preparation protocols (Additional file 1: Figure S10). These artefacts can be detrimental to downstream analysis as the sequencing library partially represents artificial fragments.

**Figure 4:**
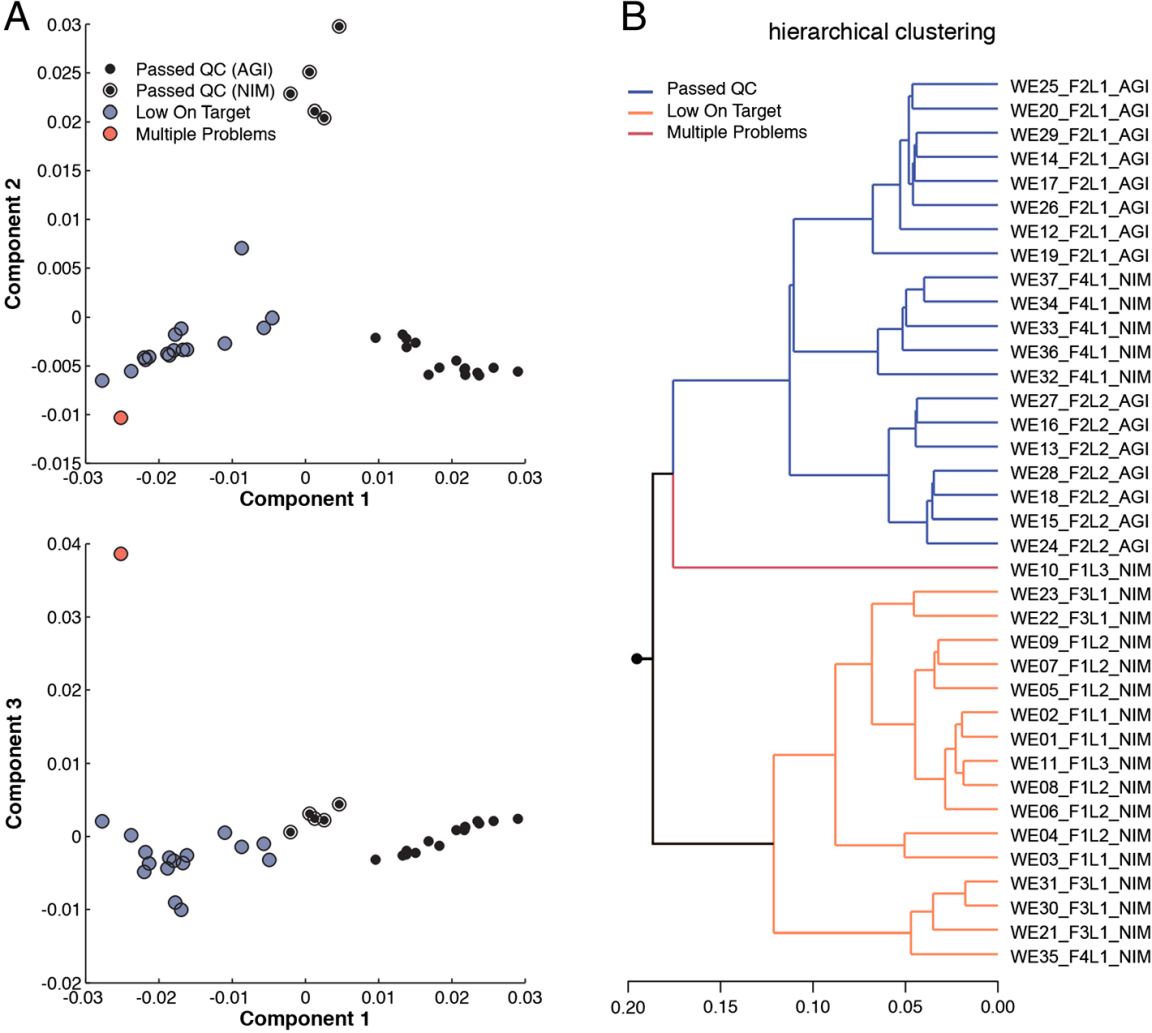
***k*** **-mer distances in whole exome sequencing data are associated with data quality and choice of capture protocol. (A)** PCA of pairwise distance measures. Blue circles indicate samples with poor capture performance. The red circles highlight the WE10_F1L3_NIM sample, which suffers from multiple problems. Samples that passed the QC measures are indicated by different types of black circle based on the choice of capture kit (Nimblegen or Agilent SureSelect). **(B)** Hierarchical clustering of pairwise *k*-mer distance measures across WES samples. Different clusters are indicated by color. AGI, Agilent SureSelect; NIM, Nimblegen; PCA, principal component analysis; QC, quality control; WES, whole exome sequencing.

In WES datasets, we identified four clusters after applying principal component analysis (PCA) on the distances obtained from a pairwise comparison of smoothed 12-mers (Figure 4A). Principal component 1 (PC1) separated samples based on the rate of on-target reads (reads that map to the exons for which probes were designed). The low level of reads on target is the result of poor capture performance and not of low sequencing depth (Additional file 1: Figure S11A,B). Interestingly, PC2 separates the successful WES datasets (69.9% on-target reads, on average) based on the type of capture kit (Agilent or Nimblegen) that was used during the library preparation (Figure 4A). The third principal component separates out a single failed dataset, WE10_F1L3_NIM. This dataset has multiple problems since the rate of on-target reads is only 3.7% and the duplication rate is as high as 80%. The extreme level of duplication significantly affects the balance of coverage on the plus and minus strands of the reference genome. Therefore, the *k*-mer profile remains imbalanced since most *k*-mers and their reverse complements have different frequencies. While the hierarchical clustering concords with that of PCA, we observed another sub-clustering among failed samples in which samples with only 11.3% of reads on target were separated from those that exhibit an on-target rate of 49.8% (Figure 4B). The influence of poor capture performance on *k*-mer profiles is evident from the *k*-mer frequency distributions, as those with poor capture performance begin to mimic that of the full genome (Additional file 1: Figure S12A,B), due to an increase in the number of off-target reads. The multimodality of these spectra is the result of off-target reads that map to non-coding and repetitive regions [13]. Notably, samples that passed QC could be separated by the capture kit used during library preparation as a result of differences between the targeted regions of capture kits (Additional file 1: Figure S12C).

**Figure 5:**
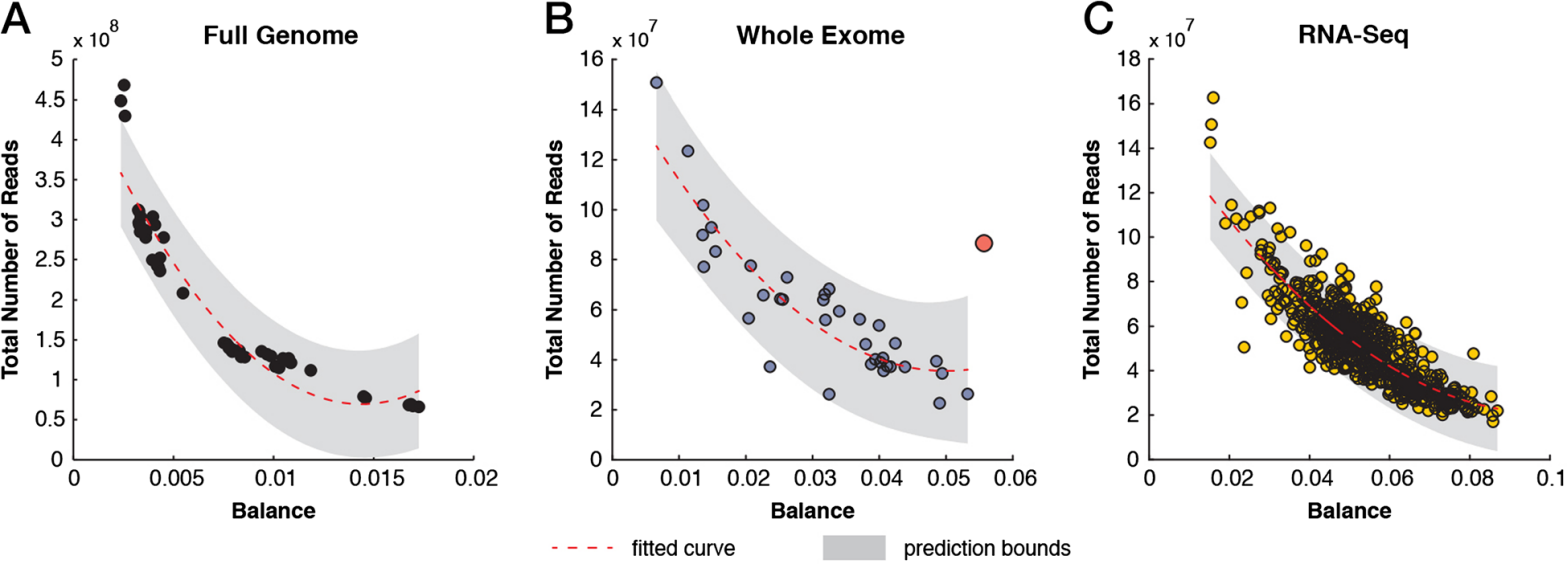
**Detecting the balance in coverage depth of plus and minus strands in sequencing data. (A)** Scatter plot of distance between the frequencies of *k*-mers and their reverse complement (balance) versus the total number of reads in WGS data. The poly2 fitted line and the 95% confidence intervals are indicated. **(B)** Scatter plot of balance versus the total number of reads in WES data. The red circle indicates an outlier with an extreme duplication rate and imbalance of coverage between the plus and minus strands. **(C)** Scatter plot of balance versus the total number of reads in RNA-Seq data. RNA-Seq, RNA sequencing; WES, whole exome sequencing; WGS, whole genome sequencing.

The analysis of balance between the frequency of *k*-mers and their reverse complement can expose library biases and provide a measure for estimating an optimal sequencing depth to ensure comparable and sufficient coverage on both strands (Additional file 1: Notes). In human WGS datasets, the balance curve begins to level off as datasets exceed 400 million reads, which represents an approximately 12-times coverage of an entire human genome (Figure 5A). Although the balance curve did not saturate in our WES set, we picked up WE10_F1L3_NIM as an outlier since the expected balance distance is roughly 0.015 for datasets with a comparable number of reads (Figure 5B). This sample suffers from multiple problems. However, its extreme level of duplications (80%) contributes to the imbalanced coverage on the plus and minus strands (Additional file 1: Figure S13). In the RNA-Seq set, the change in balance begins to level off at the 140 million reads mark (Figure 5C). Of course, this approach will not hold for strand-specific RNA-Seq runs. These data can now be used to assess whether an independent sequencing run has the expected balance distance and, thus, whether sufficient sequencing depth has been achieved.

### Comparative analysis of kPAL performance

We benchmarked the performance of kPAL in the identification of problematic samples by comparing the QC analysis of kPAL on a subset of WGS, WES and RNA-Seq samples with results from the *Preqc* function of the recently developed *k*-mer based String Graph Assembler (SGA) [22]. SGA can estimate genome size, insert size distribution, repeat content and heterozygosity of a sequenced genome as well as the error rate and its potential consequence in *de novo* assembly. Unlike kPAL, SGA does not perform a pairwise comparison between *k*-mer profiles obtained from multiple datasets. Thus, we compared SGA’s performance to that of kPAL based on the identification of known problematic samples, using SGA’s estimated genome size, fragment size distribution and the overall error rate. A further evaluation of SGA on the selected datasets is presented in Additional file 1: Figures S14–S17.

In WGS data from the first sample (FG1), SGA confirmed the bimodal insert size distribution of libraries that were prepared based on the first protocol (Additional file 1: Figure S15). Moreover, sequencing data from the two library preparation protocols could be separated based on the position of the first occurring sequencing errors (Additional file 1: Figure S14A). This is in concordance with kPAL results and the presence of a higher level of library chimeras that led to the introduction of artificial and rare *k*-mers.

The selected WES data consists of two samples with failed capture (WE01_F1L1_NIM and WE02_F1L1_NIM), one sample with multiple problems (WE10_F1L3_NIM), and four samples with acceptable quality that were prepared using Agilent or Nimblegen capture kits (WE13_F2L2_AGI, WE14_F2L1_AGI, WE36_F4L1_NIM and WE37_F4L1_NIM). SGA identified the problematic sample WE10_F1L1_NIM, which suffers from an extremely high duplication rate and a very low number of on-target reads (Additional file 1: Figure S14B). The estimated genome size or duplication rate did not further assist in identifying problematic samples and the position of the first sequencing error seems to be obscured by the low coverage of off-target reads that may resemble erroneous sequences. Together, identification of problematic samples by SGA is less reliable for WES data than whole genome shotgun sequences.

For RNA-Seq data, we selected two samples that passed all quality measures (HG00096.1 and HG00108.7) and four failed samples with different underlying problems (HG00329.5: high duplication; NA12546.1: high rRNA; NA18858.1: poor alignment and NA18861.4: high genomic DNA contamination). SGA’s genome size estimation is designed for WGS data and, therefore, applying SGA on RNA-Seq data should provide an estimate of the expressed part of the genome. Genomic DNA contamination artificially increases the expressed part of the genome and allowed SGA to identify NA18861.4 as a problematic sample (Additional file 1: Figure S14C). SGA could not reliably identify HG00329.5 as a sample with an exceptionally high duplication rate (Additional file 1: Figure S14C). Unlike kPAL, the SGA analysis could not identify the other problematic RNA-Seq samples.

### Detecting data complexity

**Figure 6:**
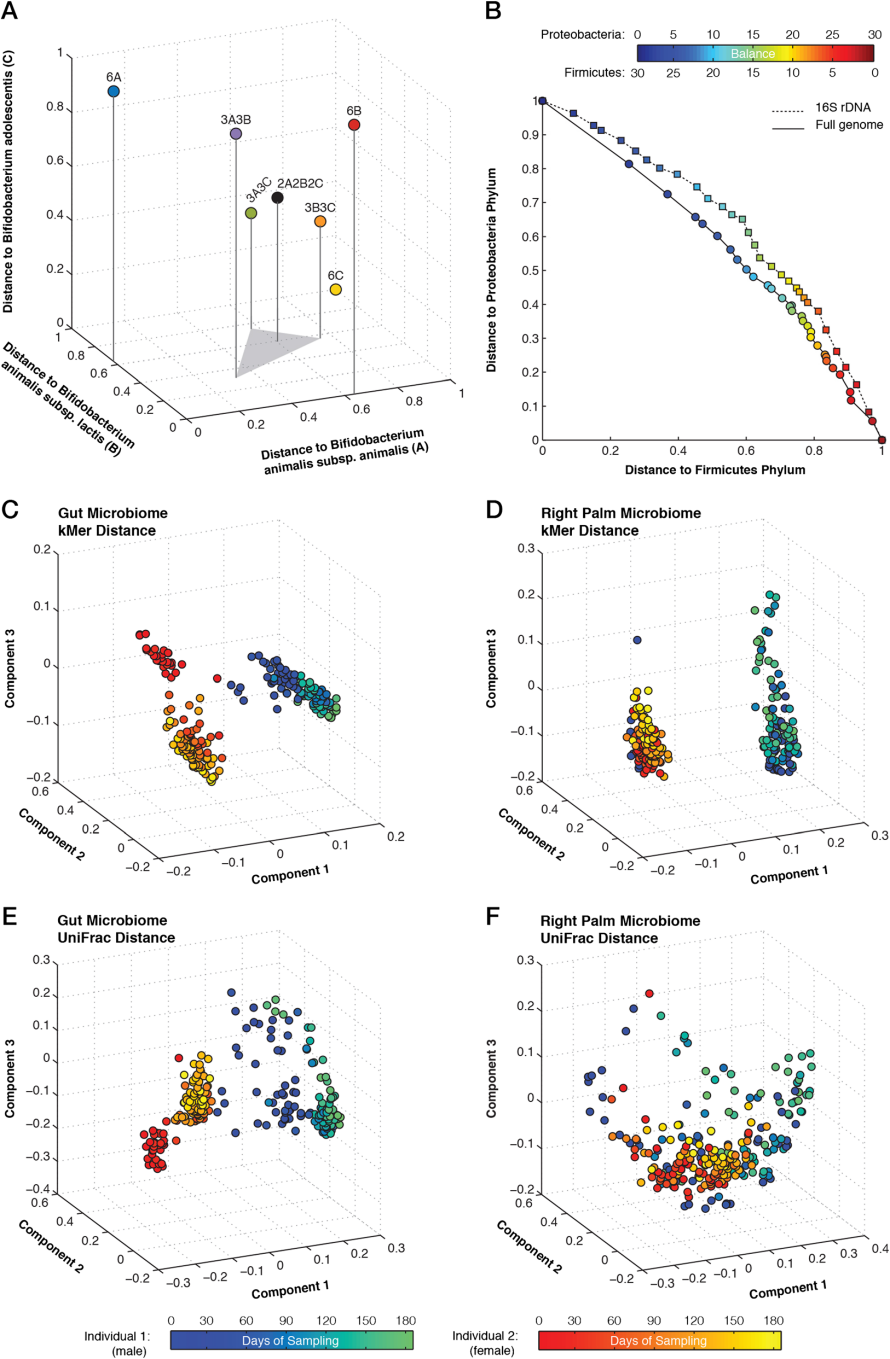
**Resolving the level of relatedness between microbiomes. (A)** Three-dimensional scatter plot of the *k*-mer distance measures for a series of metagenomes with different copy number of three closely related species. **(B)** Scatter plot of the relative distance between Firmicutes and Proteobacteria phyla. Each data point represents a metagenome with a differing number of species from each phylum. Data points are colored according to the number of species from each phylum. **(C)** PCA plot of pairwise *k*-mer distance measures for gut microbiomes. Data points are colored based on the origin of the sample (male in blue and female in red) and time. **(D)** PCA plot of pairwise *k*-mer distance measures for right-palm microbiomes. **(E)** PCA plot of pairwise UniFrac distance measures for gut microbiomes. **(F)** PCA plot of pairwise UniFrac distance measures for right-palm microbiomes. PCA, principal component analysis.

The complexity of sequencing libraries is reflected in the *k*-mer spectrum as *k*-mer frequencies often represent functional or structural elements of the associated genome. For metagenomes, the abundance of different bacteria diversifies the frequency of *k*-mers, which can be used to differentiate microbiome communities. To investigate the application of kPAL in the comparative analysis of microbiomes, we first simulated a series of metagenomes with different copy number for three closely related bacterial genomes: *Bifidobacterium animalis* subspecies *lactis* (NC_017834.1), *Bifidobacterium animalis* subspecies *animalis* (NC_017867.1) and *Bifidobacterium adolescentis* (NC_008618.1). The selected genomes have a comparable genome size of approximately 2 Mbp. The level of homology between *Bifidobacterium animalis* subspecies *lactis* and *Bifidobacterium animalis* subspecies *animalis* is estimated to be between 85% and 95% [27]. The genomes of these bacteria are represented in copies of 6:0:0, 3:3:0 and 2:2:2. The distances from a pairwise comparison of 10-mer profiles show an interesting pattern (Figure 6A). Within the three-dimensional space of individual species, datasets with six copies of a single genome lie within a main triangular space bounded by the absolute minimum distance to their corresponding species. The second triangular space holds datasets that have three copies of two genomes while the dataset with two copies of all genomes sits in the middle of the three-dimensional space (Figure 6A). The relatedness of these datasets relies on the number of rare *k*-mers that could differentiate the abundance of different species within each set.

Next, we explored the capability of kPAL in resolving the composition of a more complex series of simulated metagenomes. Without considering the phylogeny, 30 bacterial genomes were selected from both the Firmicutes and Proteobacteria phyla and used to construct 31 datasets where the first set comprises 30 genomes from the Firmicutes phylum. The sequence content of each set was subsequently shifted to the Proteobacteria phylum by single genome substitutions (Additional file 1: Table S2). Thus, the 31st dataset consists of 30 genomes from only the Proteobacteria phylum. After performing the pairwise distance comparison on 10-mer profiles, datasets were plotted based on their distance to each phylum (Figure 6B). Notably, the order of the datasets concords with the number of genomes from each phylum. Although the modelled metagenomes do not reflect the true relative abundance of these bacteria, they allow us to assess whether kPAL can resolve the level of similarity between a series of modelled metagenomes. Distances between *k*-mer profiles generated on the 16S rDNA also confirm the relative similarity of datasets with a slightly smoother transition. This is mainly due to the limited amount of genomic information that is available in 16S rDNA and different rate of evolution compared to the entire genome.

We used the previously published data by Caporaso *et al*. [28] to evaluate further the performance of kPAL in resolving microbiomes. The gut and right-palm microbiomes of a male individual and a female individual were sequenced over a period of 6 months. For this analysis, we only included samples that were collected on the same day from both individuals (122 gut microbiomes and 128 right-palm microbiomes). Furthermore, we also excluded 14 samples that were classified as being mislabeled using a random forest classifier as described by Caporaso *et al*. [28]. Pairwise distances were calculated for samples from each body part using kPAL (using 10-mer profiles) and UniFrac [29], which relies on the characterization of operational taxonomic units and inferred phylogeny. UniFrac parameters were set to those specified in the original paper [28]. The agreement between the expected clusters (based on the origin of samples) and that obtained from distance matrices was estimated using the weighted kappa index (*Kw*). PCA analysis of *k*-mer distance matrices from gut (Figure 6C) and right-palm (Figure 6D) microbiomes revealed that samples from each individual could be separated using the kPAL approach (*Kw* = 0.95 and 0.82, respectively). In addition, PC2 and PC3 indicate that temporal changes in the microbiomes of each individual influence the relative distances between datasets. We also noticed that datasets from the first 12 days of right-palm microbiomes from the male individual cluster with female samples. This can be caused by possible contamination or sample swapping. Gut microbiomes could also be resolved using UniFrac (Figure 6E), with *Kw* = 0.94. Concordant to the kPAL results, PC2 and PC3 jointly order samples based on the sampling day. However, UniFrac failed to differentiate right-palm microbiomes based on their origin (*Kw* = 0.47) with no apparent pattern corresponding to the day on which samples were collected (Figure 6F).

## Conclusions

The continued decrease in sequencing costs and technological development have overtaken our ability to assess the quality of data and the complexity of sequencing libraries robustly. For instance, many QC steps that are essential for accurate downstream analysis of NGS data are often neglected in the absence of a reliable reference genome. In addition, NGS data are always subjected to some degree of technical and run-to-run variation, which can hamper the interpretation of the genetic makeup of the sequenced sample. As shown here, variations introduced during library preparation can have a significant influence on the complexity and quality of the sequencing data.

So far, *k*-mer profiles have been used in a wide range of applications, such as the identification of regulatory elements, error correction of sequencing reads, identification of point mutations, whole genome assembly, searches for homologous regions and phylogenetic analysis [15-21,30,31]. A number of *k-mer* analysis tools are capable of efficiently generating *k*-mer profiles (such as Jellyfish [32] and khmer [33]), and the recent work of Simpson [22] proposes a novel method to estimate the repeat content, genome size, heterozygosity of the sequenced genome, insert size distribution and estimated level of erroneous reads in sequencing data using a *k*-mer approach. Although SGA provides valuable information on the genetic makeup and quality of sequencing data, it cannot reliably identify outliers from a series of NGS data or provide information on potential sources of variation. Thus, in the absence of a well-characterized reference sequence, there is an urgent need for tools that can characterize potential biases such as sample swapping, library chimeras, high duplication rates and potential contamination. In this work, we introduce a new strategy for determining the quality and complexity of a variety of different NGS datasets without any prior information about the reference sequence. The kPAL package consists of a variety of tools to generate *k*-mer frequencies and enables pairwise comparisons. kPAL measures the level of similarity between multiple NGS datasets, based on the genomic information that is shared between them. We show that kPAL outperforms pre-alignment QC tools (such as FastQC) in reliably exposing samples that suffer from poor capture performance, contamination, enrichment of library chimeras or other types of artefact. Even though the last step in assessing data quality by FastQC involves the analysis of overrepresented 5-mers, FastQC fails to identify problematic samples due to the low *k*-mer size and the way *k*-mer profiles are processed. In contrast, tools that rely on aligned reads (such as RNA-SeQC [34] and the Picard toolkit) can expose the majority of these technical artefacts, though some of them still require a thorough and vigorous assessment to be identified. The *Preqc* feature of SGA performs well on WGS data and can precisely estimate insert size distribution and expose erroneous reads. However, the performance of SGA on other types of NGS data, such as WES and RNA-Seq, is less reliable since it was originally developed for pre-processing, error correction and *de novo* assembly of whole genome sequences. The lack of a pairwise comparison and accommodation for differences in library size limits the application of SGA in quality assessment and measuring the level of dissimilarity between *k*-mer profiles of sequenced samples. The unique feature of kPAL is its ability to account for biases introduced by differences in sequencing depth between samples to expose outliers and problematic samples and that, like SGA, it does not rely on prior information. Potential applications of this strategy are to determine the quality of sequencing data, estimate the sequencing depth required for *de novo* assembly projects and identifying sequencing reads that represent the uncharacterized regions of the genome of a given species.

Most microbiome studies have focused on phylogenetically informative markers such as 16S rDNA to reveal the relative composition and diversity of the metagenome in question (reviewed in [7,35]). Despite the efficiency of such approaches, amplicon-based studies lack the ability to provide a genome-wide characterization of microbiomes. Moreover, sequencing errors and the presence of library chimeras can hamper the analysis of microbiomes using conventional tools, as only a handful of reads may be produced from any given fragment. This results in unreliable operational taxonomic units, which are often used in microbiome studies. The advantage of our approach is that it can potentially discriminate between different species of a common phylum by relying on sequence content beyond the resolution of 16S rDNA sequences. We show that the similarity of microbiomes based on their composition and diversity can be revealed using kPAL, which is purely founded upon the sequencing data alone. In contrast, although UniFrac could reliably resolve rather stable gut microbiomes, it struggled with resolving highly diverse and dynamic microbiomes, such as those obtained from skin (i.e., the palm). We show that kPAL is sensitive to temporal changes in microbiomes and can potentially be used for a wide range of applications, such as forensic DNA fingerprinting. It is important to note that further developments are required for reliable assessment of temporal changes in a microbial community using the kPAL approach. Although kPAL does not provide a biological reason for the sources of variation within and between datasets, it opens the way to a more accurate and unbiased determination of the quality and complexity of genomic sequences.

## Materials and methods

### kPAL implementation

kPAL is a Python-based toolkit and programming library that provides various tools, many of which are used in this study. kPAL is an open-source package and can be downloaded [36-38]. kPAL can also be installed (including all prerequisites) through the command line using: pip install kPAL. Detailed documentation and tutorials are available [39]. For detailed a description of the kPAL methodology, refer to Additional file 1: Notes. The performance of kPAL, in terms of speed and memory usage, for generating and pairwise comparison of *k*-mer profiles is provided in Additional file 1: Figure S18.

### Creating *k*-mer profiles

The *k*-mer profiles were generated using the *index* function built into kPAL. For all analyses *k* was set to 12 except when otherwise stated. To accommodate for the analysis of both sequencing reads and genome reference sequences, we have chosen to use the FASTA format as an input to kPAL. However, we provide a command-line tool to convert FASTQ files to the appropriate format [40]. For paired-end data, the profiles for both reads were merged into a single *k*-mer profile using the kPAL *merge* function. For more information on performance, runtime and memory usage, see Additional file 1: Notes.

### Measuring pairwise distances

The *matrix* function was used in combination with the *scale* and/or *smooth* options to measure the distance between two *k*-mer profiles. The pairwise distance between profiles was calculated using the multiset distance measure [41]. This measure was parameterized by a function that reflects the distance between two elements in a multiset, in this case the difference between frequencies of specific *k*-mers. The following function was used to calculate the distances after applying the *scale* and *smooth* options.

$$ f\left(x,y\right)=\frac{\left|x-y\right|}{\left(x+1\right)\left(y+1\right)} $$

For further information about the procedure, refer to Additional file 1: Notes.

### Calculating the *k*-mer balance

For all samples in this study, the balance between the frequencies of *k*-mers and their reverse complement were found using the *showbalance* function in kPAL (see Additional file 1: Notes). For all paired-end datasets, *k*-mer profiles were first merged and then assessed for their balance.

### Statistical analysis

The distance matrices produced by the pairwise comparison of all samples were used to perform a hierarchical clustering and PCA in R and MATLAB, respectively. The mRNA analysis pipeline, QC and exon quantification procedure are described elsewhere [23,24]. For the microbiomes, the hierarchical clustering was done using the distance matrices provided by the *k*-mer profile or UniFrac [29] analyses. Subsequently, the accuracy of the clustering arrangement was assessed based on the silhouette [42] and weighted kappa [43] measures.

### Library preparation and sequencing

For WGS datasets, two separate library preparation protocols were used. The gDNA libraries for full genome libraries were prepared using the reagents from a TrueSeq DNA Sample Prep Kit according to the manufacturer’s instructions (TrueSeq DNA Sample Preparation Guide, revision C; Illumina Inc., San Diego, CA) with minor modifications. After the ligation, the first protocol uses a gel-free method for samples instead of a gel step that was used for the second protocol. Furthermore, the number of PCR cycles in the PCR enrichment step differs between the two protocols (five and ten cycles, respectively). A High Sensitivity DNA chip (Agilent Technologies 2100; Santa Clara, CA) was used for quantification and samples were subsequently sequenced on an Illumina HiSeq 2000 sequencer at the same laboratory.

Libraries for the WES samples were prepared using the Agilent SureSelect Kit (Agilent Technologies, Santa Clara, CA), Nimblegen Capture Kit V2 or Nimblegen Capture Kit V3 (Roche NimbleGen Inc., Madison, WI), according to the manufacturer’s instructions. A High Sensitivity DNA chip (Agilent Technologies 2100) was used for the quantification and the samples were subsequently sequenced on an Illumina HiSeq 2000 sequencer at the same laboratory.

The library preparation and sequencing of all RNA-Seq samples are described elsewhere [23,24].

### Pre-processing

FastQC was run for all samples prior to analysis to assess the quality of the data. However, none of the sequencing data was removed from the analysis as they all passed the FastQC quality measures. Reads were trimmed for low quality bases (*Q* < 20) using sickle [44] and cleaned up for adapters.

### Alignment

Alignment to the human reference genome was performed for WGS and WES using Stampy [45], BWA [46] and Bowtie 2 [47] with default parameters. For the WES samples, the number of on-target reads was calculated using the BEDTools [48] intersect, BAM files and a BED track consisting of all targets according to the manufacturer’s guidelines. Reads with no overlapping base were considered as off target. Basic alignment statistics (such as alignment rate, the fraction of properly paired reads, etc.) were extracted using SAMtools [49] flagstat. For WGS samples, the insert sizes were estimated using the Picard toolkit [50]. The number of base pairs that were soft clipped during the alignment was extracted from the SAM files using a custom script.

### SGA comparison

QC and exploration of data properties were performed using the *Preqc* module of the SGA software. All analyses were performed according to SGA guidelines [22].

### Data availability

For the WGS and WES data, the FASTQ and BAM files have been deposited at the European Genome-phenome Archive [51], which is hosted by the European Bioinformatics Institute, under the accession number [EGA:S00001000600]. In addition, all *k*-mer profiles are available under the same accession.

For the RNA-Seq data, the *k*-mer profiles can be found online [52]. The FASTQ files and BAM alignments as well as different types of quantification are available in ArrayExpress under accessions E-GEUV-1 (mRNA) and E-GEUV-2 (small RNA) for QC-passed samples and E-GEUV-3 for all sequenced samples [53-55].

Microbiomes were obtained from the ‘Moving Pictures of the Human Microbiome’ project [MG-RAST:4457768.3-4459735.3] [28].

## Additional file

Additional file 1:
**Supplemental notes, figures and tables.**

## Abbreviations

kPAL: *k*-mer Profile Analysis Library
Mbp: Megabase pairs
NGS: Next-generation sequencing
PCA: Principal component analysis
PCR: Polymerase chain reaction
QC: Quality control
RNA-Seq: RNA sequencing
SGA: String Graph Assembler
UTR: Untranslated region
WES: Whole exome sequencing
WGS: Whole genome sequencing
