# Supplementary material for: Determining the quality and complexity of next-generation sequencing data without a reference genome
Source: Genome Biol. 2014 Dec 17;15(12):555. doi: 10.1186/s13059-014-0555-3 (PMC4298064; doi:10.1186/s13059-014-0555-3)
Supplement: Additional file 1: — Supplemental notes, figures and tables. [file 13059_2014_555_MOESM1_ESM.pdf]

Determining the quality and complexity of next-generation sequencing data without a reference genome

Seyed Yahya Anvar[1,2,*], Lusine Khachatryan[1], Martijn Vermaat[2], Michiel van Galen[2], Irina Pulyakhina[1], Yavuz Ariyurek[2], Ken Kraaijeveld[2,3], Johan T. den Dunnen[1,2], Peter de Knijff[1], Peter A.C. 't Hoen[1,4], Jeroen F.J. Laros[1,2,4,*]

[1] Department of Human Genetics and [2] Leiden Genome Technology Center, Leiden University Medical Center, Leiden, the Netherlands
[3] Department of Ecological Science, Section Animal Ecology, VU University Amsterdam, Amsterdam, the Netherlands
[4] Netherlands Bioinformatics Centre, Leiden, the Netherlands.

∗ To whom correspondence should be addressed:

SYA Tel: 0031715268559; Email: s.y.anvar@lumc.nl
JFJL Tel: 0031715269504; Email: j.f.j.laros@lumc.nl

Supplementary Notes

The distribution of k-mers (DNA words of length k) in sequencing data can provide a unique view of the complexity and quality. It has been shown that features that occur too often or too rarely reflect crucial information about the functional and structural elements of the genome of the sequenced species (1-5). Thus, the number of overrepresented sequence motifs does not disappear by increasing the k size. Many studies have shown the unimodality of the genomic k-mer spectra of most species with the exception of mammalians (1). All mammalians exhibit a multimodal distribution of k-mer frequencies. This feature highlights the existence of common and extremely rare features that reflect the complexity of the genome in question. The multimodality of the k-mer spectrum is independent of the genome size. Chor et al. (1) have shown that while the genome of *T. thermophila* has a comparable size (97Mb) to that of the human chromosome 12 (108Mb), the k-mer spectra differ in modality (unimodal and multimodal, respectively). The modality of the human genome is also subjected its function. Strikingly, all coding regions, including the 5' un-translated regions (UTRs) exhibit a unimodal k-mer spectrum while the introns, 3' UTRs and other intergenic regions hold a multimodal distribution (1,3). Moreover, the composition of k-mers in the spectrum is context-specific. Nullomers (missing k-mers) and rare k-mers tend to be GC-rich and often contain many CpGs (1,5). This is caused by the hypermutability of CpGs (C → T and G → A; also known as CpG suppression) that mutate at 10-20 times higher rate than other types of point mutations (5-8). In this work, we present the utility of k-mer spectrum in determining the quality and complexity of next-generation sequencing data that relies on shared and unique features across species as shown for estimating the level of relatedness between microbiomes.

kMer Methodology

Index

The first step in any k-mer analysis is the generation of a profile (Figure 1A), which is constructed by the *indexing* algorithm. The efficiency of the algorithm is improved by encoding the DNA string in binary following the map given in Figure 1B. Subsequently, the binary encoded k-mers are used as the index of a count table. This can be achieved by the concatenation of the binary code for each nucleotide in a given DNA string. This procedure eliminates the need to store the actual k-mer sequences since they can be retrieved from decoding the offset in the count table. The binary code for each nucleotide is chosen in such a way that the complement of the nucleotide can be calculated using the binary NOT operator. The *indexing* algorithm returns a profile that holds observed counts for all possible substrings of length k that can be stored for other analyses.

Distance (*diff*)

Since the k-mer profile is in essence a vector of almost independent values, we can use any metric defined for vectors to calculate the *distance* between two profiles. We have implemented two metrics which are the standard Euclidian distance measure and the *multiset* (9) distance measure (see Formula 1). The last metric is parameterised by a function that reflects the difference between a pair. We have implemented two pairwise distance functions (shown in Formulae 2 and 3).

For a multiset X, let $S(X)$ denote its underlying set. For multisets X, Y with $S(X), S(Y) \subseteq \{1, 2, \ldots, n\}$ we define:

(1)

$$d_f = \frac{\sum_{i=1}^{n} f(x_i, y_i)}{|S(X) \cup S(Y)| + 1}$$

(2) Pairwise function:

$$f_1(x, y) = \frac{|x - y|}{(x + 1)(y + 1)}$$

(3) Pairwise function:

$$f_2(x, y) = \frac{|x - y|}{x + y + 1}$$

Balance (*showbalance*)

When analysing sequencing data, which frequently consist of reads from both strands (e.g., due to non strand-specific sample preparation or paired-end sequencing), we can assume that the chance of observing a fragment originating from the plus and minus strands are equal. Additionally, if the sequencing depth is high enough, we expect a *balance* between the frequencies of

k-mers and their reverse complement in a given k-mer profile. Every type of NGS data has an expected balance (i.e., SAGE is not expected to produce a balanced profile while whole genome shotgun sequencing is expected to have a perfectly balanced frequency between k-mers and their reverse complement). Thus, k-mer balance can indicate the quality of NGS data in respect to over-amplification, insufficient number of reads, or poor capture performance in the case of whole exome sequencing.

To calculate the balance, first we observe that every k-mer has a reverse complement. One of these is lexicographically smaller (or equal in the case of a *palindrome*) than the other. We first split a profile into two vectors, $A = (a_0, a_1, \dots)$ and $B = (b_0, b_1, \dots)$ where b_i represents the reverse complement of a_i and vice versa. The distance between these vectors can be calculated in the same way as described for pairwise comparison of two full k-mer profiles (Figure 1C).

Additionally, kMer can forcefully balance the k-mer profiles (if desired) by adding the values of each k-mer to its reverse complement. This procedure can improve distance calculation if the sequencing depth is too low.

Shrink

A profile indexed at a certain k size contains information about k-mers of smaller lengths. This can be seen from the fact that a word w over an alphabet $\mathcal{A}$ has $|\mathcal{A}|$ possible suffixes of length one. To calculate the number of occurrences of w, we simply need to calculate the $\sum_{i \in \mathcal{A}} count(w \,.\, i)$. This only holds when k is relatively small compared to the length of the indexed sequencing reads. Indeed, if a sequence of length ℓ is indexed at length k, then $(\ell - k + 1)$ k-mers are encountered per sequence. However, shrinking of a profile will yield $(\ell - k)$ k-mers. Usually, this border effect is small enough to ignore, but should be taken into consideration when indexing large amounts of small (approaching length k) sequences. Shrinking is useful when trying to estimate the best k for a particular purpose. One can start with choosing a relatively large k and then reuse the generated profile to construct a profile of smaller k sizes (Figure 1D).

Smoothing

Ideally, the samples that are used to generate profiles are sequenced with the same sample preparation, on the same platform, and most importantly at sufficient depth. However, in practice, this is rarely the case. When two similar samples are sequenced at insufficient depth, it will be reflected in a k-mer profile by zero counts for k-mers that are not expected to be nullomers. While this is not a problem in itself, the fact that most sequencing procedures have a random selection of sequencing fragments will result in a random distribution of these zero counts. When comparing two profiles, the pairwise distances will be artificially large. Scaling the profiles can partially compensate for differences in the sequencing depth but can not account for nullomers since no distinction can be made between true missing words and artificially missing words. An obvious solution would be to shrink the profile until nullomers are removed. This method is valid as long as all zero counts reflect artificial nullomers. Otherwise, shrinking will reduce the specificity and does not reflect the true complexity of the sequenced genome. To deal with this problem, we have developed the *pairwise smoothing* function. This method locally shrinks a profile only when necessary. In this way, we retain information if it is available in both profiles and discard missing data (Figure 1E).

Let P and Q be sub-profiles of words over an alphabet $\mathcal{A}$ of length ℓ (with ℓ devidable by $|\mathcal{A}|$). Let t be a user-defined threshold and let f be a method of summarizing a profile. If $\min(f(P), f(Q)) > t$ we divide the profiles in $|\mathcal{A}|$ equal parts and recursively repeat the procedure for each part. If this is not the case, we collapse both P and Q to one word. Implemented methods of summarizing are minimum, mean, and median. In Figure 1E we show an example of how smoothing might work. We have chosen $f = min$ and $t = 0$ as default parameters. With this method, we can index a dataset with a large k and retain the overall specificity of the profile since this method can automatically select the optimal choice of k locally.

Below, we provide an overview of all functions of kMer that are available via the command line interface:

Function	Description
index	Make a profile from a FASTA file
merge	Merge two profiles
balance	Balance a profile on the frequency of k-mers and their reverse complements
showbalance	Calculate the balance of a profile
meanstd	Show the mean and standard deviation of k-mer frequencies
distr	Calculate the distribution of the frequencies in a profile
info	Print basic statistics on a given profile
getcount	Retrieve the count for a particular k-mer
positive	Only keep counts that are positive in both profiles
scale	Scale profiles such that the total number of k-mer frequencies is equal
shrink	Shrink a profile, effectively educing k size
shuffle	Randomise a profile
smooth	Smooth two profiles by collapsing sub-profiles
diff	Calculate the distance between two profiles
matrix	Make a pairwise distance matrix for a series of k-mer profiles

Supplementary Table 1 – An overview and basic statistics on NGS data.

ID	Protocol Code	Total Reads	Alignment (%)	Duplication (%)	Prop. Pairs (%)	Discordant (%)	On Target (%)
**Whole Genome Sequencing (WGS) data**							
FG1_F4L1_P2	Protocol 2	446654194	99.88	2.85	95.85	2.04	NA
FG1_F4L2_P2	Protocol 2	243872592	99.87	2.01	95.89	2.03	NA
FG1_F1L1_P1	Protocol 1	117691658	99.59	2.27	83.41	13.53	NA
FG1_F1L2_P1	Protocol 1	122955824	99.59	2.35	83.38	13.56	NA
FG1_F1L3_P1	Protocol 1	108288914	99.59	2.11	83.45	13.50	NA
FG2_F4L2_P2	Protocol 2	199745404	99.87	1.85	95.07	2.69	NA
FG2_F4L3_P2	Protocol 2	411392680	99.87	3.00	95.03	2.71	NA
FG2_F1L4_P1	Protocol 1	129056860	99.61	1.84	85.26	11.89	NA
FG2_F1L5_P1	Protocol 1	127011660	99.63	1.81	85.28	11.87	NA
FG2_F1L6_P1	Protocol 1	132618524	99.63	1.87	85.27	11.89	NA
FG3_F1L7_P1	Protocol 1	118035394	99.56	1.94	84.18	12.84	NA
FG3_F1L8_P1	Protocol 1	122261090	99.60	2.03	84.24	12.81	NA
FG3_F4L4_P2	Protocol 2	428887478	99.88	2.89	96.09	1.71	NA
FG4_F2L2_P1	Protocol 1	129062580	99.72	1.74	87.46	9.93	NA
FG4_F2L3_P1	Protocol 1	123029712	99.70	1.69	87.46	9.92	NA
FG4_F2L4_P1	Protocol 1	122774394	99.67	1.70	87.39	9.90	NA
FG4_F2L2_P2	Protocol 2	288710022	99.86	2.30	95.40	2.04	NA
FG4_F2L3_P2	Protocol 2	280581930	99.86	2.27	95.39	2.05	NA
FG4_F2L4_P2	Protocol 2	270864574	99.85	2.25	95.38	2.04	NA
FG5_F2L5_P2	Protocol 2	285364738	99.87	1.90	96.53	1.59	NA
FG5_F2L6_P2	Protocol 2	275417304	99.86	1.87	96.47	1.61	NA
FG5_F2L7_P2	Protocol 2	279423494	99.86	1.93	96.49	1.59	NA
FG5_F2L5_P1	Protocol 1	133079914	99.76	1.84	91.87	5.95	NA
FG5_F2L6_P1	Protocol 1	129077350	99.76	1.82	91.82	5.98	NA
FG5_F2L7_P1	Protocol 1	131237400	99.75	1.83	91.89	5.91	NA
FG6_F3L1_P2	Protocol 2	296508646	99.71	2.58	95.25	2.56	NA
FG6_F3L2_P2	Protocol 2	286834464	99.70	2.54	95.25	2.56	NA
FG6_F2L8_P2	Protocol 2	271555116	99.85	2.52	95.44	2.52	NA
FG6_F3L1_P1	Protocol 1	65996740	99.23	1.68	87.50	9.59	NA
FG6_F3L2_P1	Protocol 1	65390164	99.14	1.67	87.41	9.60	NA
FG6_F2L8_P1	Protocol 1	63421100	99.69	1.66	87.91	9.64	NA
FG7_F3L3_P2	Protocol 2	302013082	99.82	2.19	96.18	1.81	NA
FG7_F3L4_P2	Protocol 2	295235714	99.82	2.16	96.18	1.81	NA
FG7_F3L5_P2	Protocol 2	303851942	99.83	2.18	96.20	1.81	NA
FG7_F3L3_P1	Protocol 1	108774848	99.56	1.83	81.72	15.32	NA
FG7_F3L4_P1	Protocol 1	107269844	99.56	1.81	81.70	15.34	NA
FG7_F3L5_P1	Protocol 1	109243670	99.58	1.83	81.75	15.30	NA
FG8_F3L6_P2	Protocol 2	243965640	99.80	1.68	95.97	2.13	NA
FG8_F3L7_P2	Protocol 2	278758454	99.80	1.82	95.96	2.13	NA
FG8_F3L8_P2	Protocol 2	287101398	99.79	1.84	95.96	2.12	NA
FG8_F3L6_P1	Protocol 1	64415080	99.61	1.07	87.47	9.99	NA
FG8_F3L7_P1	Protocol 1	73986900	99.60	1.19	87.44	10.00	NA
FG8_F3L8_P1	Protocol 1	75888242	99.54	1.21	87.43	9.98	NA
FG9_F5L5_P2	Protocol 2	234767462	99.84	1.96	95.98	1.84	NA
FG9_F5L6_P2	Protocol 2	236372176	99.83	1.97	95.98	1.84	NA
FG9_F5L7_P2	Protocol 2	230093402	99.83	1.93	95.96	1.85	NA
FG9_F5L5_P1	Protocol 1	140145696	99.72	1.96	90.57	6.94	NA
FG9_F5L6_P1	Protocol 1	141026172	99.71	1.96	90.57	6.93	NA
FG9_F5L7_P1	Protocol 1	137203932	99.71	1.92	90.55	6.94	NA
**Whole Exome Sequencing (WES) data**							
WE01_F1L1_NIM	Nimblegen	83259226	99.41	30.79	87.69	6.44	4.10
WE02_F1L1_NIM	Nimblegen	77084010	99.45	20.22	91.95	3.65	4.54
WE03_F1L1_NIM	Nimblegen	68285448	99.55	63.90	87.85	6.62	4.25
WE04_F1L2_NIM	Nimblegen	56284428	99.48	58.44	85.26	7.95	5.15
WE05_F1L2_NIM	Nimblegen	64156718	99.42	58.02	92.53	3.49	5.79
WE06_F1L2_NIM	Nimblegen	77582290	99.34	56.53	89.79	4.97	5.74
WE07_F1L2_NIM	Nimblegen	56579026	99.39	33.44	91.55	4.03	5.75
WE08_F1L2_NIM	Nimblegen	89899166	99.47	33.03	92.37	3.59	5.37
WE09_F1L2_NIM	Nimblegen	64436078	99.47	52.28	89.41	5.51	5.80
WE10_F1L3_NIM	Nimblegen	86560130	99.33	80.35	77.81	12.22	3.69
WE11_F1L3_NIM	Nimblegen	92930912	99.55	41.64	91.81	4.29	5.73
WE12_F2L1_AGI	Agilent	37132998	99.76	25.64	99.26	0.13	73.91
WE13_F2L2_AGI	Agilent	59463000	99.79	38.79	99.12	0.16	72.78
WE14_F2L1_AGI	Agilent	39026800	99.71	11.10	99.25	0.14	73.00
WE15_F2L2_AGI	Agilent	66249084	99.7	33.87	99.13	0.18	72.34
WE16_F2L2_AGI	Agilent	38263608	99.79	14.96	99.18	0.19	71.47
WE17_F2L1_AGI	Agilent	35598044	99.74	10.23	99.25	0.14	72.56
WE18_F2L2_AGI	Agilent	55952210	99.79	13.36	99.29	0.11	72.25
WE19_F2L1_AGI	Agilent	22641360	99.73	8.38	98.99	0.12	72.75

WE20_F2L1_AGI	Agilent	37199940	99.77	14.16	99.12	0.37	73.73
WE21_F3L1_NIM	Nimblegen	150724496	98.98	12.60	91.74	4.40	32.29
WE22_F3L1_NIM	Nimblegen	37265636	98.99	43.41	90.65	0.84	40.41
WE23_F3L1_NIM	Nimblegen	26184756	98.97	17.19	92.89	1.77	42.40
WE24_F2L2_AGI	Agilent	72841656	99.78	16.13	99.21	0.20	71.46
WE25_F2L1_AGI	Agilent	37493898	99.72	16.51	99.06	0.21	74.07
WE26_F2L1_AGI	Agilent	40715928	99.75	22.49	98.99	0.42	72.24
WE27_F2L2_AGI	Agilent	46221708	99.75	24.41	99.22	0.17	73.91
WE28_F2L2_AGI	Agilent	63970074	99.75	26.20	99.24	0.14	72.82
WE29_F2L1_AGI	Agilent	40170162	99.75	15.01	99.08	0.10	73.22
WE30_F3L1_NIM	Nimblegen	101770558	99.42	20.03	96.31	0.39	61.51
WE31_F3L1_NIM	Nimblegen	123446142	99.38	15.90	96.73	0.37	62.20
WE32_F4L1_NIM	Nimblegen	26271934	99.48	18.76	96.14	2.19	62.16
WE33_F4L1_NIM	Nimblegen	39446618	99.4	38.43	96.53	1.50	59.55
WE34_F4L1_NIM	Nimblegen	46648798	99.35	28.97	96.83	0.93	62.13
WE35_F4L1_NIM	Nimblegen	65861096	99.47	18.77	93.99	2.65	43.11
WE36_F4L1_NIM	Nimblegen	34564376	99.02	39.88	95.63	1.70	58.61
WE37_F4L1_NIM	Nimblegen	53798832	99.39	28.24	97.36	1.05	62.07

RNA-Seq data

Please refer to ‘t Hoen et al. and Lappalainen et al. for the detailed list of statistics (10,11).

Supplementary Table 2 – An overview of simulated metagenomes. The accession number of genomes that constitute every simulated metagenome is listed. The number of genomes from *Firmicutes* or *Proteobacteria* phyla is indicated.

	**Relative Abundance**										
Firmicutes	**0**	**1**	**2**	**3**	**4**	**5**	**6**	**7**	**8**	**9**	**10**
Proteobacteria	**30**	**29**	**28**	**27**	**26**	**25**	**24**	**23**	**22**	**21**	**20**
**Genomes**	NC_009648	NC_009636	NC_004578	NC_004129	NC_002942	NC_002696	NC_017664	NC_017663	NC_017660	NC_017651	NC_017631
	NC_009636	NC_004578	NC_004129	NC_002942	NC_002696	NC_017664	NC_017663	NC_017660	NC_017651	NC_017631	NC_017628
	NC_004578	NC_004129	NC_002942	NC_002696	NC_017664	NC_017663	NC_017660	NC_017651	NC_017631	NC_017628	NC_017625
	NC_004129	NC_002942	NC_002696	NC_017664	NC_017663	NC_017660	NC_017651	NC_017631	NC_017628	NC_017625	NC_017623
	NC_002942	NC_002696	NC_017664	NC_017663	NC_017660	NC_017651	NC_017631	NC_017628	NC_017625	NC_017623	NC_017244
	NC_002696	NC_017664	NC_017663	NC_017660	NC_017651	NC_017631	NC_017628	NC_017625	NC_017623	NC_017244	NC_017223
	NC_017664	NC_017663	NC_017660	NC_017651	NC_017631	NC_017628	NC_017625	NC_017623	NC_017244	NC_017223	NC_017171
	NC_017663	NC_017660	NC_017651	NC_017631	NC_017628	NC_017625	NC_017623	NC_017244	NC_017223	NC_017171	NC_017168
	NC_017660	NC_017651	NC_017631	NC_017628	NC_017625	NC_017623	NC_017244	NC_017223	NC_017171	NC_017168	NC_017160
	NC_017651	NC_017631	NC_017628	NC_017625	NC_017623	NC_017244	NC_017223	NC_017171	NC_017168	NC_017160	NC_016147
	NC_017631	NC_017628	NC_017625	NC_017623	NC_017244	NC_017223	NC_017171	NC_017168	NC_017160	NC_016147	NC_016027
	NC_017628	NC_017625	NC_017623	NC_017244	NC_017223	NC_017171	NC_017168	NC_017160	NC_016147	NC_016027	NC_015976
	NC_017625	NC_017623	NC_017244	NC_017223	NC_017171	NC_017168	NC_017160	NC_016147	NC_016027	NC_015976	NC_015733
	NC_017623	NC_017244	NC_017223	NC_017171	NC_017168	NC_017160	NC_016147	NC_016027	NC_015976	NC_015733	NC_015567
	NC_017244	NC_017223	NC_017171	NC_017168	NC_017160	NC_016147	NC_016027	NC_015976	NC_015733	NC_015567	NC_015410
	NC_017223	NC_017171	NC_017168	NC_017160	NC_016147	NC_016027	NC_015976	NC_015733	NC_015567	NC_015410	NC_011080
	NC_017171	NC_017168	NC_017160	NC_016147	NC_016027	NC_015976	NC_015733	NC_015567	NC_015410	NC_011080	NC_010995
	NC_017168	NC_017160	NC_016147	NC_016027	NC_015976	NC_015733	NC_015567	NC_015410	NC_011080	NC_010995	NC_010717
	NC_017160	NC_016147	NC_016027	NC_015976	NC_015733	NC_015567	NC_015410	NC_011080	NC_010995	NC_010717	NC_010688
	NC_016147	NC_016027	NC_015976	NC_015733	NC_015567	NC_015410	NC_011080	NC_010995	NC_010717	NC_010688	NC_010681
	NC_016027	NC_015976	NC_015733	NC_015567	NC_015410	NC_011080	NC_010995	NC_010717	NC_010688	NC_010681	NC_017173
	NC_015976	NC_015733	NC_015567	NC_015410	NC_011080	NC_010995	NC_010717	NC_010688	NC_010681	NC_017174	NC_017174
	NC_015733	NC_015567	NC_015410	NC_011080	NC_010995	NC_010717	NC_010688	NC_010681	NC_017190	NC_017190	NC_017190
	NC_015567	NC_015410	NC_011080	NC_010995	NC_010717	NC_010688	NC_010681	NC_018089	NC_018089	NC_018089	NC_018089
	NC_015410	NC_011080	NC_010995	NC_010717	NC_010688	NC_010681	NC_017195	NC_017195	NC_017195	NC_017195	NC_017195
	NC_011080	NC_010995	NC_010717	NC_010688	NC_010681	NC_013315	NC_013315	NC_013315	NC_013315	NC_013315	NC_013315
	NC_010995	NC_010717	NC_010688	NC_010681	NC_017299	NC_017299	NC_017299	NC_017299	NC_017299	NC_017299	NC_017299
	NC_010717	NC_010688	NC_010681	NC_017304	NC_017304	NC_017304	NC_017304	NC_017304	NC_017304	NC_017304	NC_017304
	NC_010688	NC_010681	NC_017295	NC_017295	NC_017295	NC_017295	NC_017295	NC_017295	NC_017295	NC_017295	NC_017295
	NC_010681	NC_002953	NC_002953	NC_002953	NC_002953	NC_002953	NC_002953	NC_002953	NC_002953	NC_002953	NC_002953
Firmicutes	**11**	**12**	**13**	**14**	**15**	**16**	**17**	**18**	**19**	**20**	**21**
Proteobacteria	**19**	**18**	**17**	**16**	**15**	**14**	**13**	**12**	**11**	**10**	**9**
**Genomes**	NC_017628	NC_017625	NC_017623	NC_017244	NC_017223	NC_017171	NC_017168	NC_017160	NC_016147	NC_016027	NC_015976
	NC_017625	NC_017623	NC_017244	NC_017223	NC_017171	NC_017168	NC_017160	NC_016147	NC_016027	NC_015976	NC_015733
	NC_017623	NC_017244	NC_017223	NC_017171	NC_017168	NC_017160	NC_016147	NC_016027	NC_015976	NC_015733	NC_015567
	NC_017244	NC_017223	NC_017171	NC_017168	NC_017160	NC_016147	NC_016027	NC_015976	NC_015733	NC_015567	NC_015410
	NC_017223	NC_017171	NC_017168	NC_017160	NC_016147	NC_016027	NC_015976	NC_015733	NC_015567	NC_015410	NC_011080
	NC_017171	NC_017168	NC_017160	NC_016147	NC_016027	NC_015976	NC_015733	NC_015567	NC_015410	NC_011080	NC_010995
	NC_017168	NC_017160	NC_016147	NC_016027	NC_015976	NC_015733	NC_015567	NC_015410	NC_011080	NC_010995	NC_010717
	NC_017160	NC_016147	NC_016027	NC_015976	NC_015733	NC_015567	NC_015410	NC_011080	NC_010995	NC_010717	NC_010688
	NC_016147	NC_016027	NC_015976	NC_015733	NC_015567	NC_015410	NC_011080	NC_010995	NC_010717	NC_010688	NC_010681
	NC_016027	NC_015976	NC_015733	NC_015567	NC_015410	NC_011080	NC_010995	NC_010717	NC_010688	NC_010681	NC_014393
	NC_015976	NC_015733	NC_015567	NC_015410	NC_011080	NC_010995	NC_010717	NC_010688	NC_010681	NC_009513	NC_009513
	NC_015733	NC_015567	NC_015410	NC_011080	NC_010995	NC_010717	NC_010688	NC_010681	NC_009617	NC_009617	NC_009617
	NC_015567	NC_015410	NC_011080	NC_010995	NC_010717	NC_010688	NC_010681	NC_009633	NC_009633	NC_009633	NC_009633
	NC_015410	NC_011080	NC_010995	NC_010717	NC_010688	NC_010681	NC_009641	NC_009641	NC_009641	NC_009641	NC_009641
	NC_011080	NC_010995	NC_010717	NC_010688	NC_010681	NC_012925	NC_012925	NC_012925	NC_012925	NC_012925	NC_012925
	NC_010995	NC_010717	NC_010688	NC_010681	NC_009697	NC_009697	NC_009697	NC_009697	NC_009697	NC_009697	NC_009697
	NC_010717	NC_010688	NC_010681	NC_017992	NC_017992	NC_017992	NC_017992	NC_017992	NC_017992	NC_017992	NC_017992
	NC_010688	NC_010681	NC_018017	NC_018017	NC_018017	NC_018017	NC_018017	NC_018017	NC_018017	NC_018017	NC_018017
	NC_010681	NC_018065	NC_018065	NC_018065	NC_018065	NC_018065	NC_018065	NC_018065	NC_018065	NC_018065	NC_018065
	NC_017950	NC_017950	NC_017950	NC_017950	NC_017950	NC_017950	NC_017950	NC_017950	NC_017950	NC_017950	NC_017950
	NC_017173	NC_017173	NC_017173	NC_017173	NC_017173	NC_017173	NC_017173	NC_017173	NC_017173	NC_017173	NC_017173
	NC_017174	NC_017174	NC_017174	NC_017174	NC_017174	NC_017174	NC_017174	NC_017174	NC_017174	NC_017174	NC_017174
	NC_017190	NC_017190	NC_017190	NC_017190	NC_017190	NC_017190	NC_017190	NC_017190	NC_017190	NC_017190	NC_017190
	NC_018089	NC_018089	NC_018089	NC_018089	NC_018089	NC_018089	NC_018089	NC_018089	NC_018089	NC_018089	NC_018089
	NC_017195	NC_017195	NC_017195	NC_017195	NC_017195	NC_017195	NC_017195	NC_017195	NC_017195	NC_017195	NC_017195
	NC_013315	NC_013315	NC_013315	NC_013315	NC_013315	NC_013315	NC_013315	NC_013315	NC_013315	NC_013315	NC_013315
	NC_017299	NC_017299	NC_017299	NC_017299	NC_017299	NC_017299	NC_017299	NC_017299	NC_017299	NC_017299	NC_017299
	NC_017304	NC_017304	NC_017304	NC_017304	NC_017304	NC_017304	NC_017304	NC_017304	NC_017304	NC_017304	NC_017304
	NC_017295	NC_017295	NC_017295	NC_017295	NC_017295	NC_017295	NC_017295	NC_017295	NC_017295	NC_017295	NC_017295
	NC_002953	NC_002953	NC_002953	NC_002953	NC_002953	NC_002953	NC_002953	NC_002953	NC_002953	NC_002953	NC_002953
Firmicutes	**22**	**23**	**24**	**25**	**26**	**27**	**28**	**29**	**30**		
Proteobacteria	**8**	**7**	**6**	**5**	**4**	**3**	**2**	**1**	**0**		
**Genomes**	NC_015733	NC_015567	NC_015410	NC_011080	NC_010995	NC_010717	NC_010688	NC_010681	NC_012778		
	NC_015567	NC_015410	NC_011080	NC_010995	NC_010717	NC_010688	NC_010681	NC_013798	NC_013798		
	NC_015410	NC_011080	NC_010995	NC_010717	NC_010688	NC_010681	NC_013316	NC_013316	NC_013316		
	NC_011080	NC_010995	NC_010717	NC_010688	NC_010681	NC_014334	NC_014334	NC_014334	NC_014334		
	NC_010995	NC_010717	NC_010688	NC_010681	NC_014335	NC_014335	NC_014335	NC_014335	NC_014335		
	NC_010717	NC_010688	NC_010681	NC_004461	NC_004461	NC_004461	NC_004461	NC_004461	NC_004461		
	NC_010688	NC_010681	NC_004557	NC_004557	NC_004557	NC_004557	NC_004557	NC_004557	NC_004557		
	NC_010681	NC_014387	NC_014387	NC_014387	NC_014387	NC_014387	NC_014387	NC_014387	NC_014387		
	NC_014392	NC_014392	NC_014392	NC_014392	NC_014392	NC_014392	NC_014392	NC_014392	NC_014392		
	NC_014393	NC_014393	NC_014393	NC_014393	NC_014393	NC_014393	NC_014393	NC_014393	NC_014393		
	NC_009513	NC_009513	NC_009513	NC_009513	NC_009513	NC_009513	NC_009513	NC_009513	NC_009513		
	NC_009617	NC_009617	NC_009617	NC_009617	NC_009617	NC_009617	NC_009617	NC_009617	NC_009617		
	NC_009633	NC_009633	NC_009633	NC_009633	NC_009633	NC_009633	NC_009633	NC_009633	NC_009633		
	NC_009641	NC_009641	NC_009641	NC_009641	NC_009641	NC_009641	NC_009641	NC_009641	NC_009641		
	NC_012925	NC_012925	NC_012925	NC_012925	NC_012925	NC_012925	NC_012925	NC_012925	NC_012925		
	NC_009697	NC_009697	NC_009697	NC_009697	NC_009697	NC_009697	NC_009697	NC_009697	NC_009697		
	NC_017992	NC_017992	NC_017992	NC_017992	NC_017992	NC_017992	NC_017992	NC_017992	NC_017992		
	NC_018017	NC_018017	NC_018017	NC_018017	NC_018017	NC_018017	NC_018017	NC_018017	NC_018017		
	NC_018065	NC_018065	NC_018065	NC_018065	NC_018065	NC_018065	NC_018065	NC_018065	NC_018065		
	NC_017950	NC_017950	NC_017950	NC_017950	NC_017950	NC_017950	NC_017950	NC_017950	NC_017950		
	NC_017173	NC_017173	NC_017173	NC_017173	NC_017173	NC_017173	NC_017173	NC_017173	NC_017173		
	NC_017174	NC_017174	NC_017174	NC_017174	NC_017174	NC_017174	NC_017174	NC_017174	NC_017174		

NC_017190	NC_017190	NC_017190	NC_017190	NC_017190	NC_017190	NC_017190	NC_017190	NC_017190
NC_018089	NC_018089	NC_018089	NC_018089	NC_018089	NC_018089	NC_018089	NC_018089	NC_018089
NC_017195	NC_017195	NC_017195	NC_017195	NC_017195	NC_017195	NC_017195	NC_017195	NC_017195
NC_013315	NC_013315	NC_013315	NC_013315	NC_013315	NC_013315	NC_013315	NC_013315	NC_013315
NC_017299	NC_017299	NC_017299	NC_017299	NC_017299	NC_017299	NC_017299	NC_017299	NC_017299
NC_017304	NC_017304	NC_017304	NC_017304	NC_017304	NC_017304	NC_017304	NC_017304	NC_017304
NC_017295	NC_017295	NC_017295	NC_017295	NC_017295	NC_017295	NC_017295	NC_017295	NC_017295
NC_002953	NC_002953	NC_002953	NC_002953	NC_002953	NC_002953	NC_002953	NC_002953	NC_002953

Please note that gut and right palm microbiomes were obtained from Caporaso et al (12).

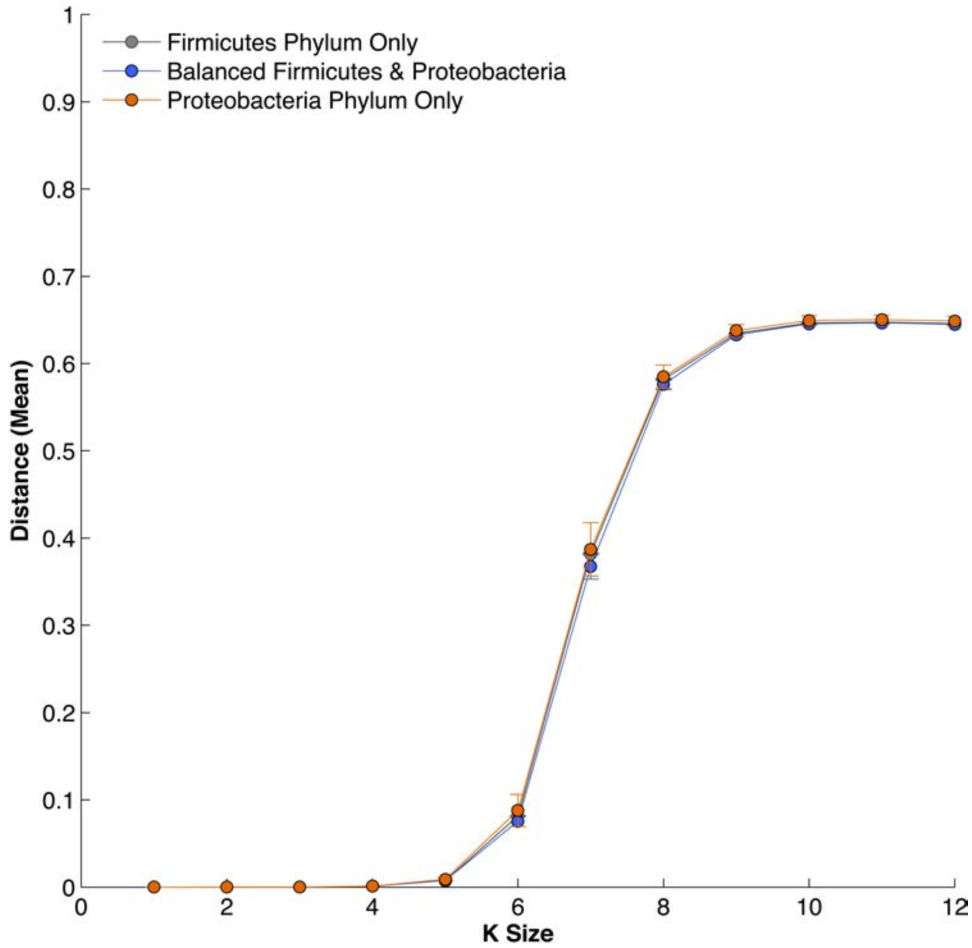

Supplementary Figure 1 – The optimal choice of *k*. The distance of three metagenomes that consist of 30 genomes from the *Firmicutes* phylum, 30 genomes selected from *Firmicutes* and *Proteobacteria* phyla, and 30 genomes from the *Proteobacteria* phylum were measured from 10 random permutations (without changing the overall nucleotide composition) based on different *k* sizes, ranging fro 1 to 12. Data points depict the median distance between each metagenome and its corresponding permutated sets. Bars indicate the standard deviation.

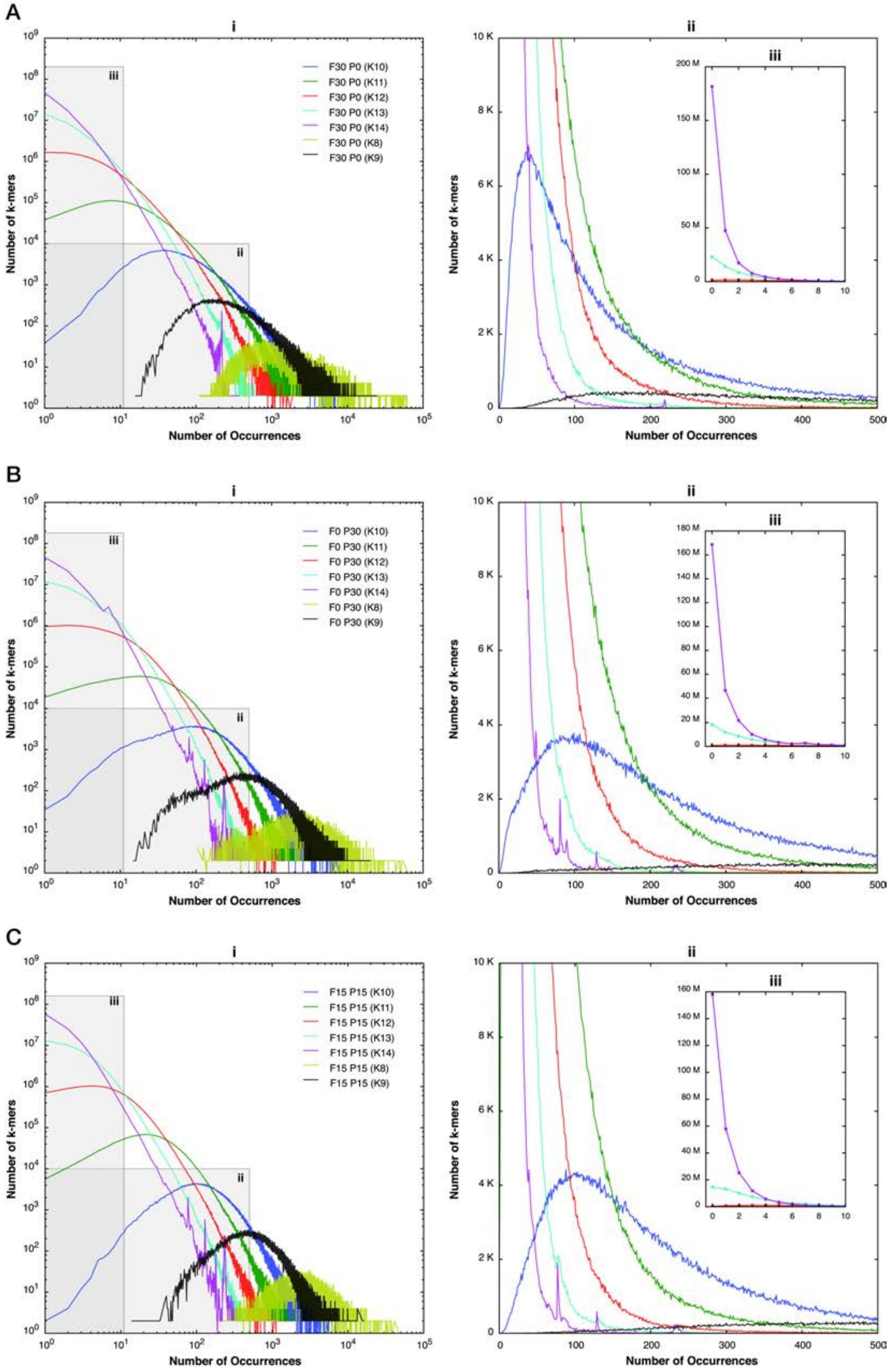

Supplementary Figure 2 – The *k*-mer spectrum of modelled metagenomes. A) The *k*-mer spectrum of metagenome consisting of 30 species from the *Firmicutes* phylum. The *k* size ranges from 8 to 14, coloured accordingly. The full spectrum is depicted in panel **i** (left panel), and two zoomed in plots (**ii** and **iii**) are provided on the right. **B)** The *k*-mer spectrum of metagenome consisting of 30 species from the *Proteobacteria* phylum. **C)** The *k*-mer spectrum of metagenome consisting of 30 species from the *Firmicutes* and *Proteobacteria* phyla.

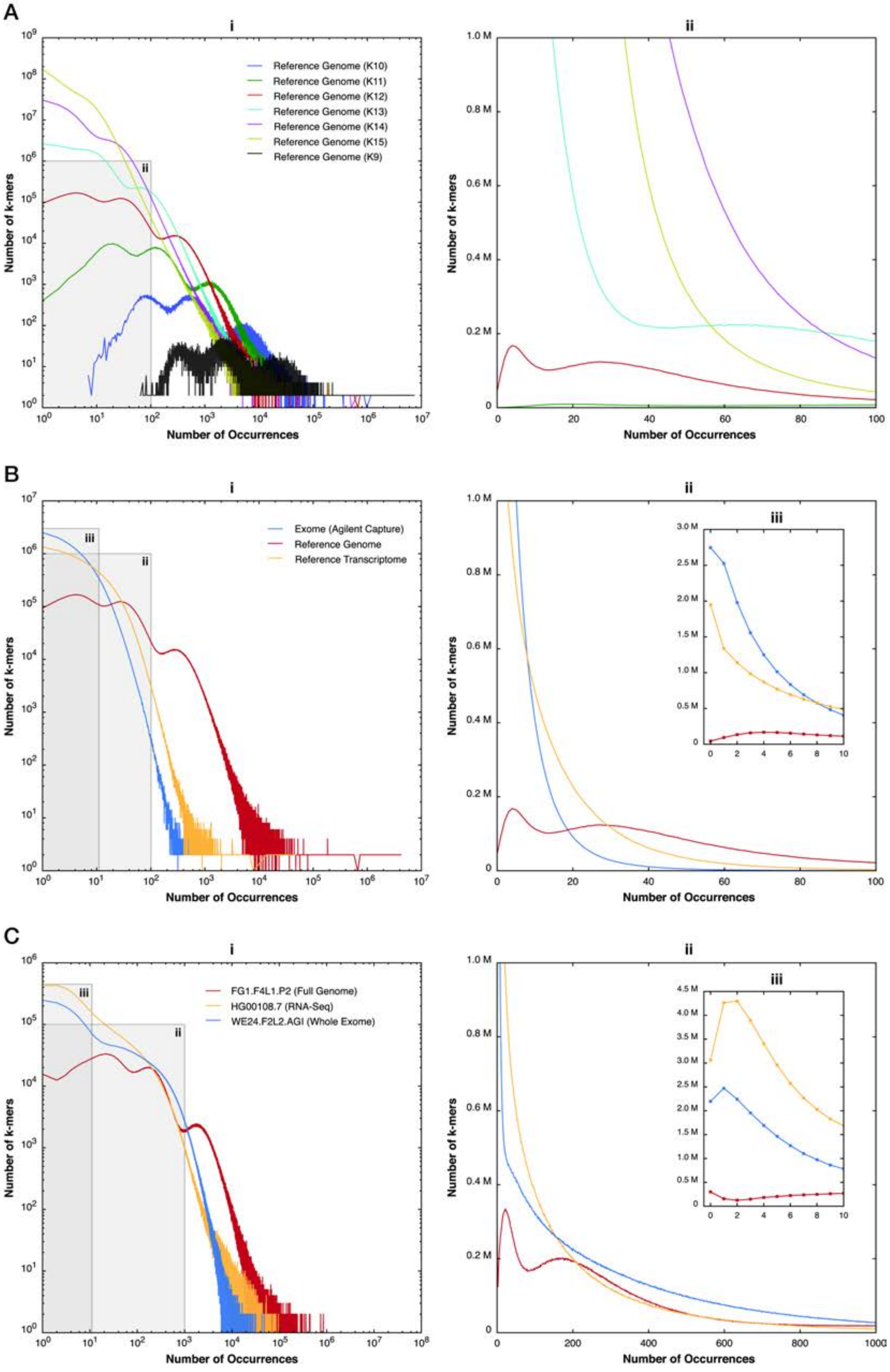

Supplementary Figure 3 – The *k*-mer spectrum on human sequencing data. A) The *k*-mer spectrum of the human reference genome (hg19) for *k* sizes ranging from 9 to 15, coloured accordingly. The full spectrum is depicted in panel **i** (left panel), and two zoomed in plots (**ii** and **iii**) are provided on the right. **B)** The *k*-mer spectrum of genome, exome, and transcriptome reference sequences. The *k* size 12 is used to generate these profiles. **C)** The *k*-mer spectrum of whole genome sequencing, whole exome sequencing and RNA-Seq data. The *k* size 12 is used to generate these profiles. Different NGS data types are indicated in different colours.

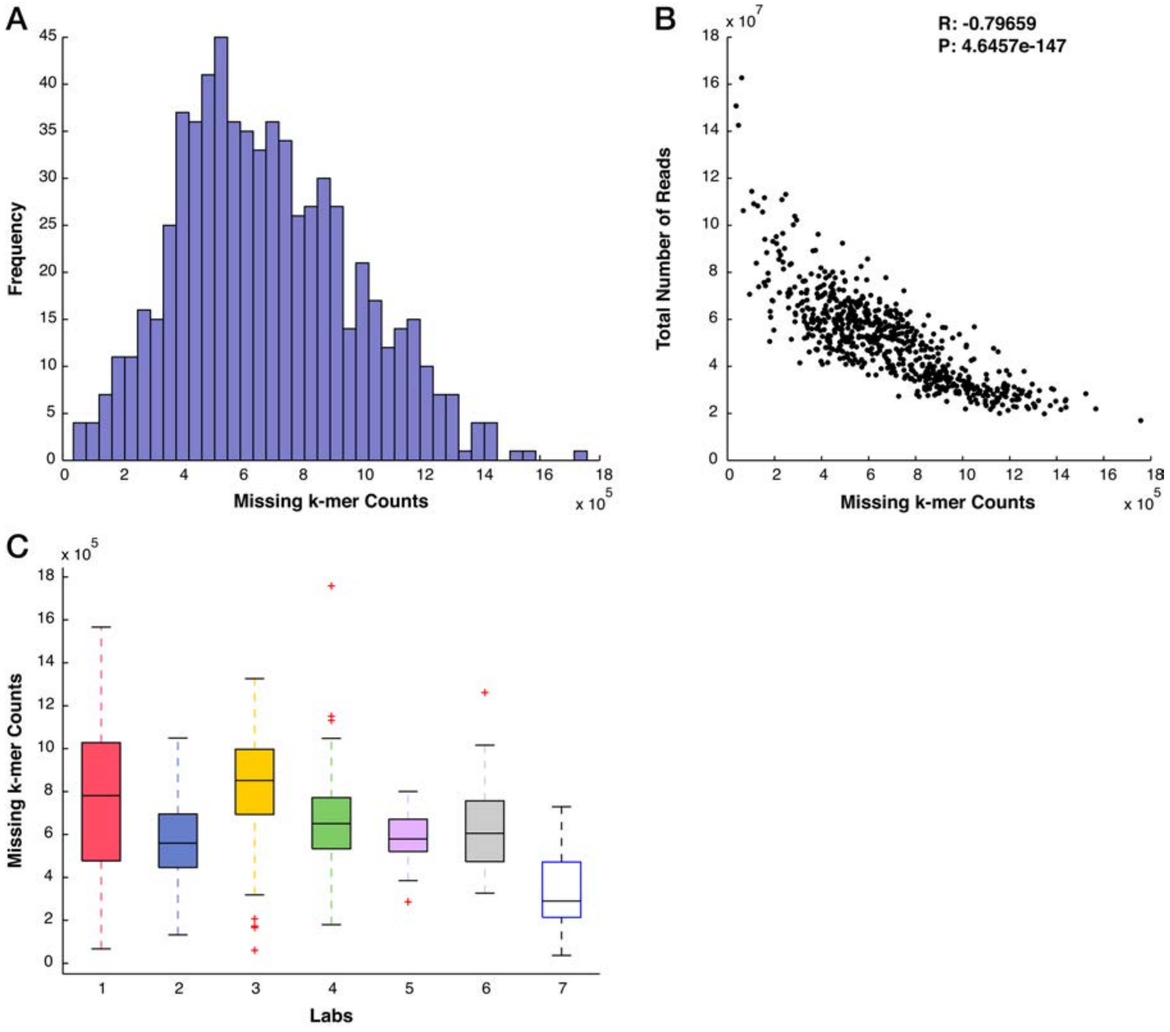

Supplementary Figure 4 – The nullomers distribution in *k*-mer spectrum of RNA-Seq data. A) Histogram of the total number of missing *k*-mers (*k*-mers with frequency of zero) across 665 RNA-Seq data. **B)** Scatter plot of the total number of missing *k*-mers versus the total number of reads per sample. **C)** Box plot of the total number of missing *k*-mers grouped according to the sequencing laboratories.

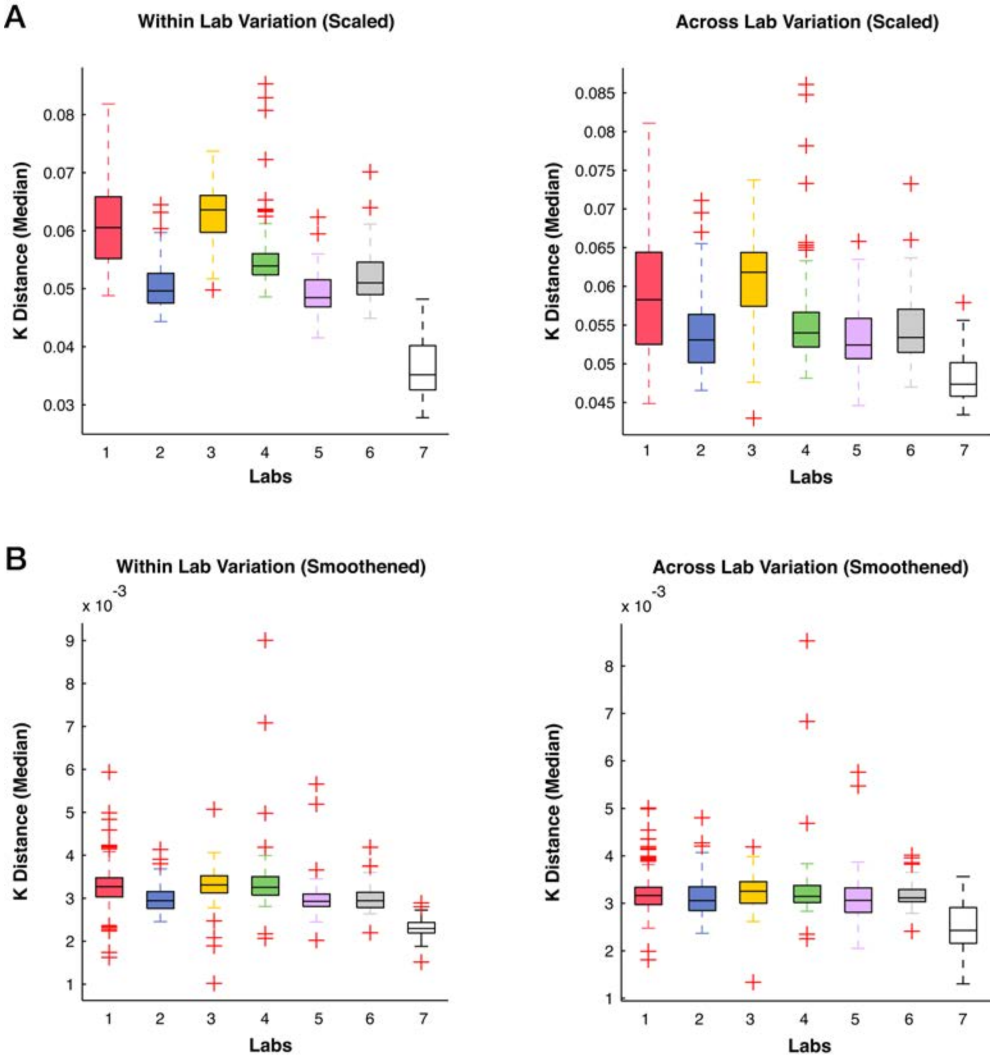

Supplementary Figure 5 – The variation between *k*-mer distances in RNA-Seq data. A) Box plots of the pairwise distance measures, **scaled only**, between samples sequenced in the same laboratory (left panel) or different laboratories (right panel). The distances are grouped for samples based on their sequencing laboratories and coloured accordingly. **B)** Box plots of the pairwise distance measures, **scaled and smoothened**, between samples sequenced in the same laboratory (left panel) or different laboratories (right panel). The distances are grouped for samples based on their sequencing laboratories and coloured accordingly.

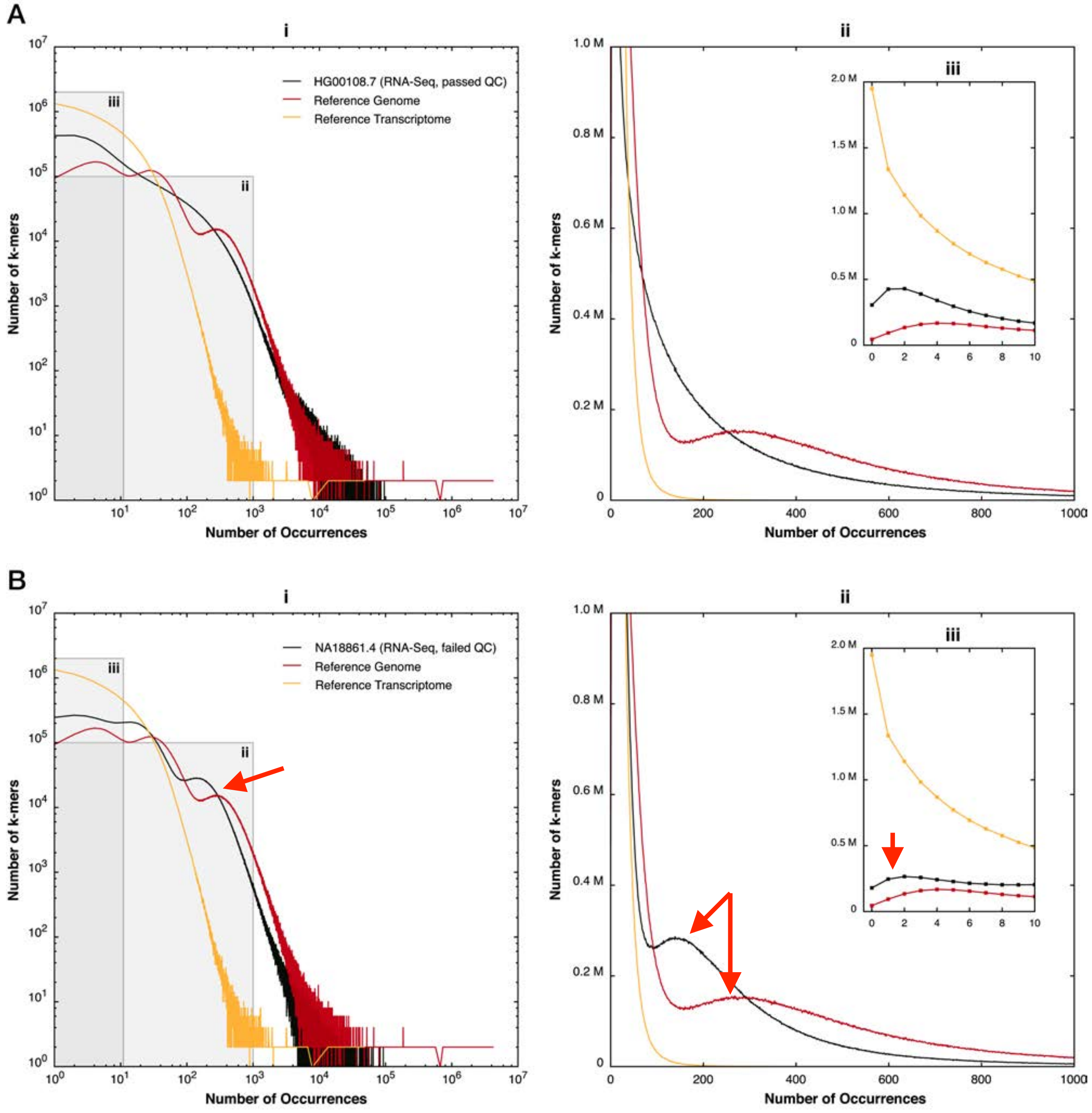

Supplementary Figure 6 – Data quality influences the complexity of the *k*-mer spectrum of RNA-Seq data. A) The *k*-mer spectrum of HG00108.7 sample that passed all QC measures for *k* size 12. The full spectrum is depicted in panel **i** (left panel), and two zoomed in plots (**ii** and **iii**) are provided on the right. **B)** The *k*-mer spectrum of NA18861.4 sample that did not pass QC measures for *k* size 12. This sample has severe contamination of genomic DNA. Both datasets have comparable number of sequencing reads.

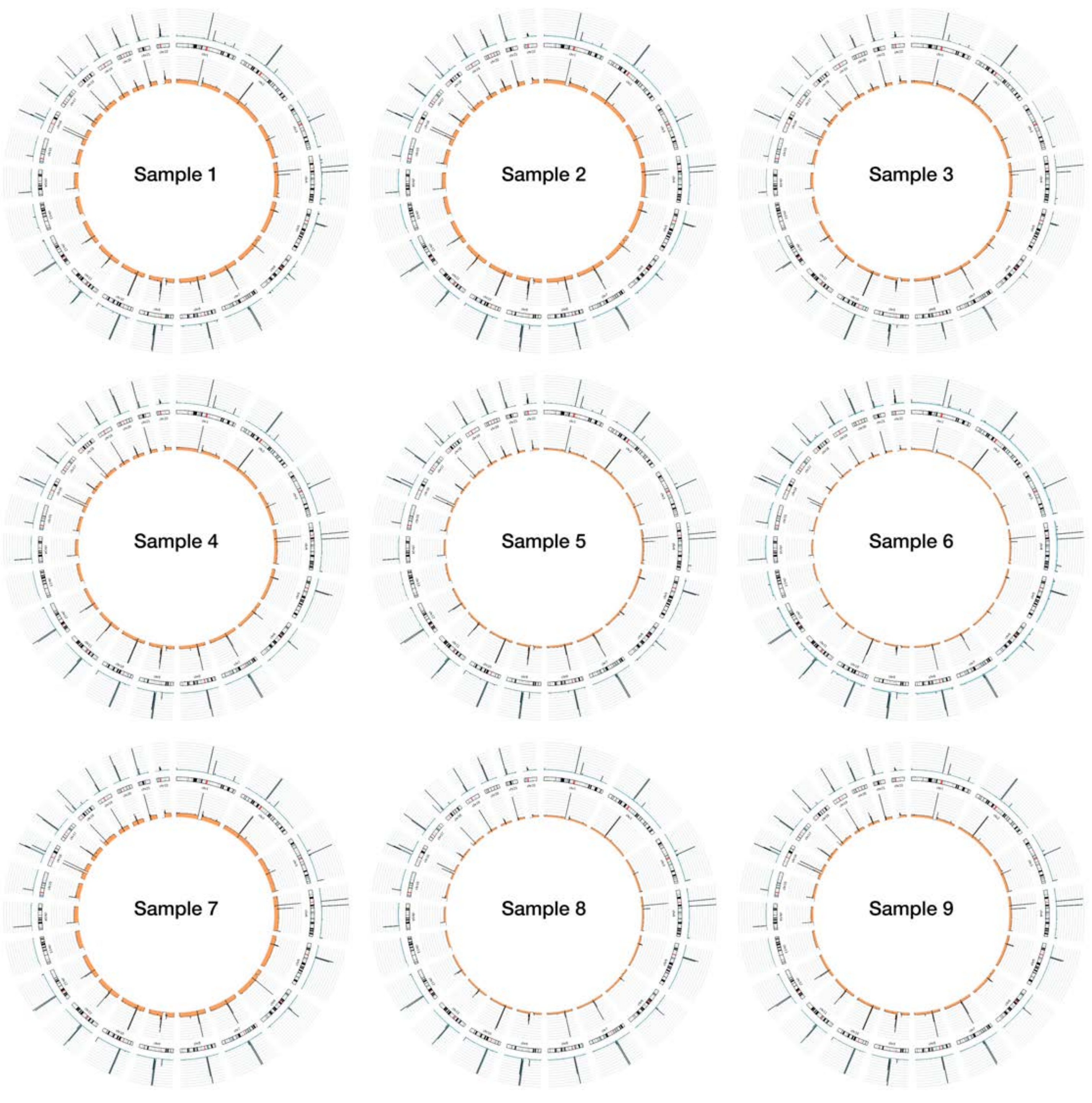

Supplementary Figure 7 – Genome-wide coverage of discordant reads in WGS data. Circos plots depict the overall coverage of discordant reads across all chromosomes for samples prepared using the first protocol (inner circles, in orange) and second protocol (outer circles, in blue). Coverage expectedly peaks at most centromeres. Datasets from each individual are merged and labelled accordingly.

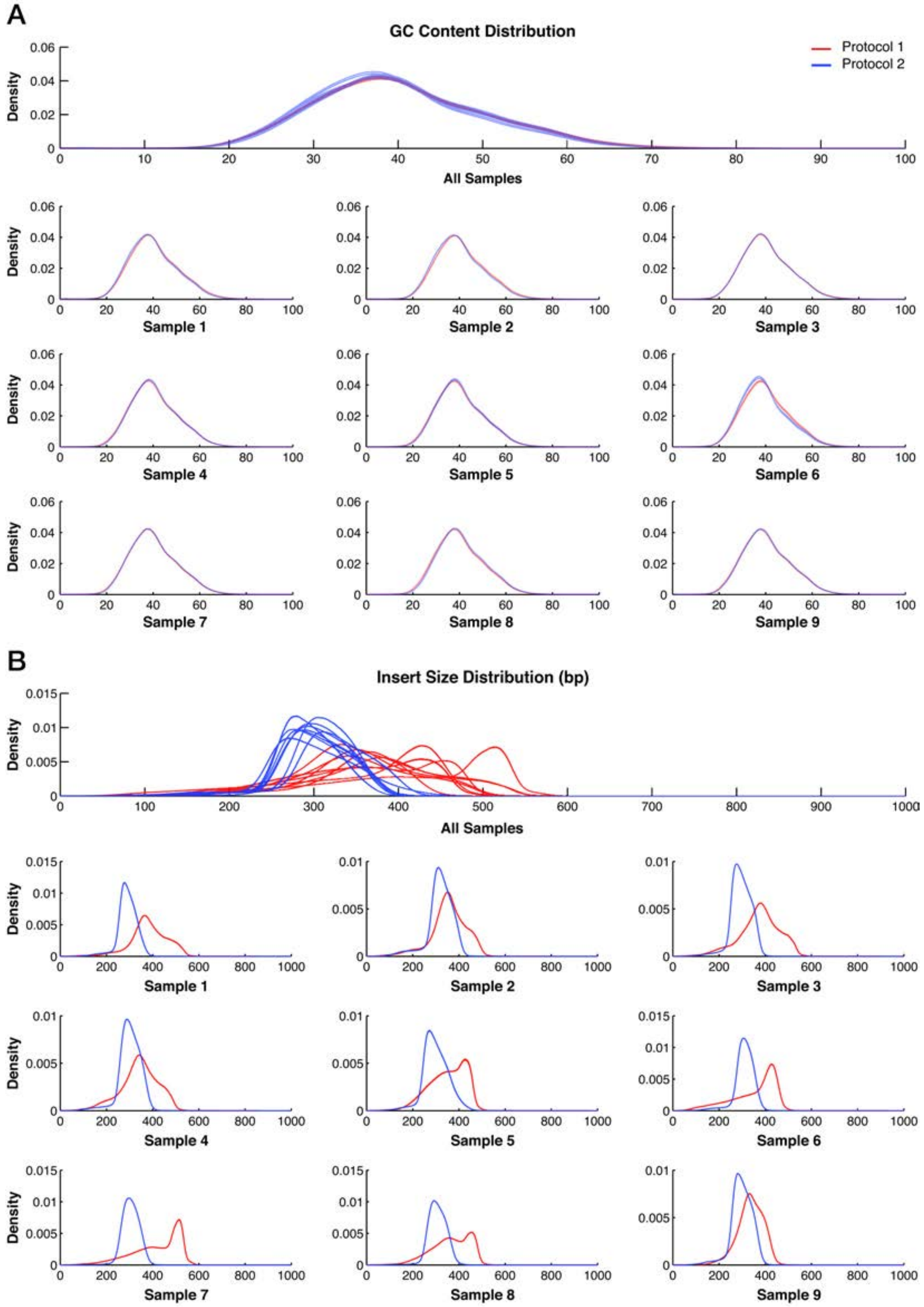

Supplementary Figure 8 – GC-content and insert size distributions in WGS data. A) The distribution of GC-content per read, grouped according to the choice of library preparation protocol (protocol one in red and protocol two in blue). Distribution for samples from each individual is provided. **B)** The distribution of the estimated insert sizes, grouped according to the choice of library preparation protocol (protocol one in red and protocol two in blue).

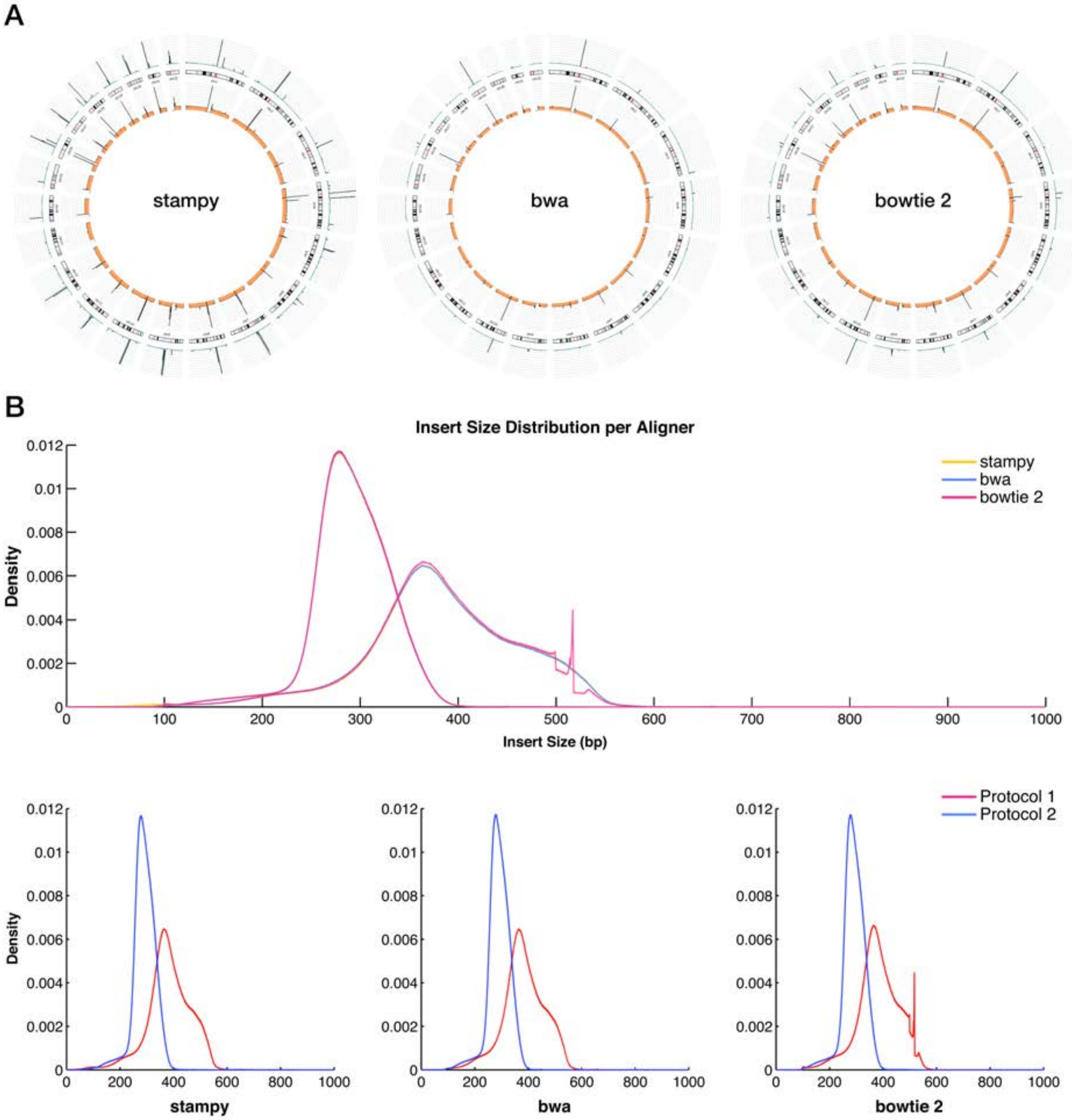

Supplementary Figure 9 – The influence of aligners in the rate of discordant reads and insert size distribution of WGS data. A) Circos plots depict the overall coverage of discordant reads across all chromosomes for samples prepared using the first protocol (inner circles, in orange) and second protocol (outer circles, in blue). Each panel provides the result obtained using different aligners (stampy, bwa, and bowtie 2, respectively). Datasets from each individual are merged and labelled accordingly. **B)** The distribution of the estimated insert sizes, grouped according to the choice of genome aligner (stampy, bwa, and bowtie 2). Lower panel shows the insert size distribution per individual aligner, coloured based on the choice of library preparation protocol (protocol one in red and protocol two in blue).

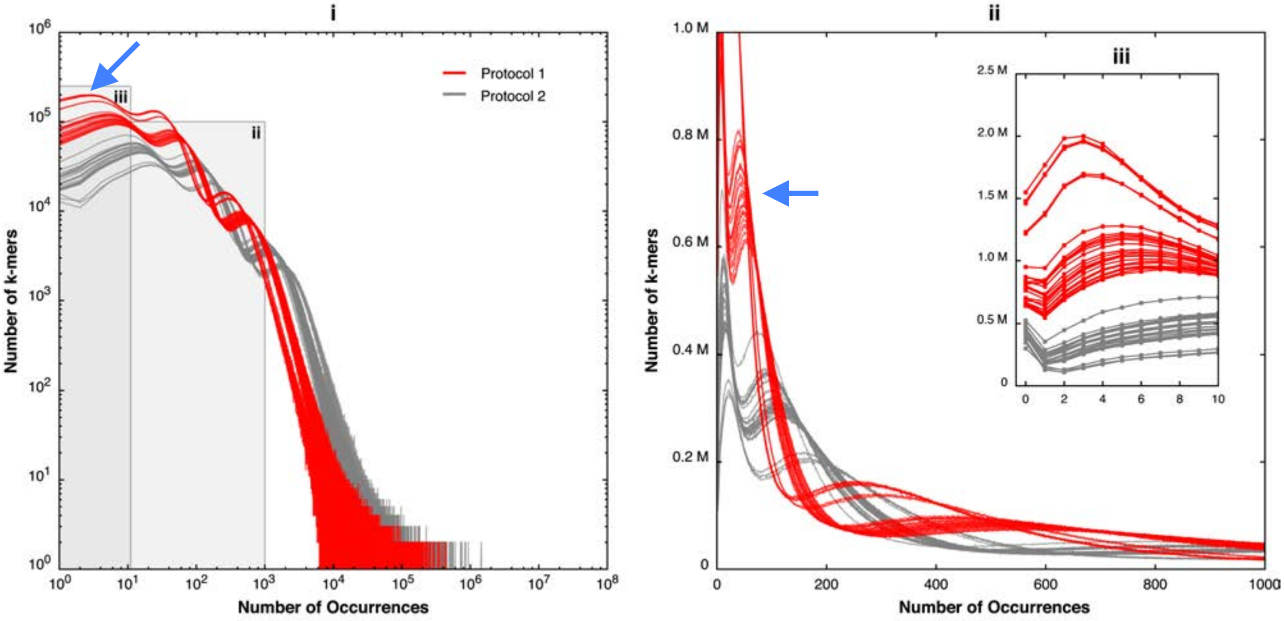

Supplementary Figure 10 – Data quality influences the complexity of the *k*-mer spectrum of WGS data. The *k*-mer spectrum (k = 12) of samples, prepared using the library preparation protocol one (red) or two (grey). The full spectrum is depicted in panel **i** (left panel), and two zoomed in plots (**ii** and **iii**) are provided on the right.

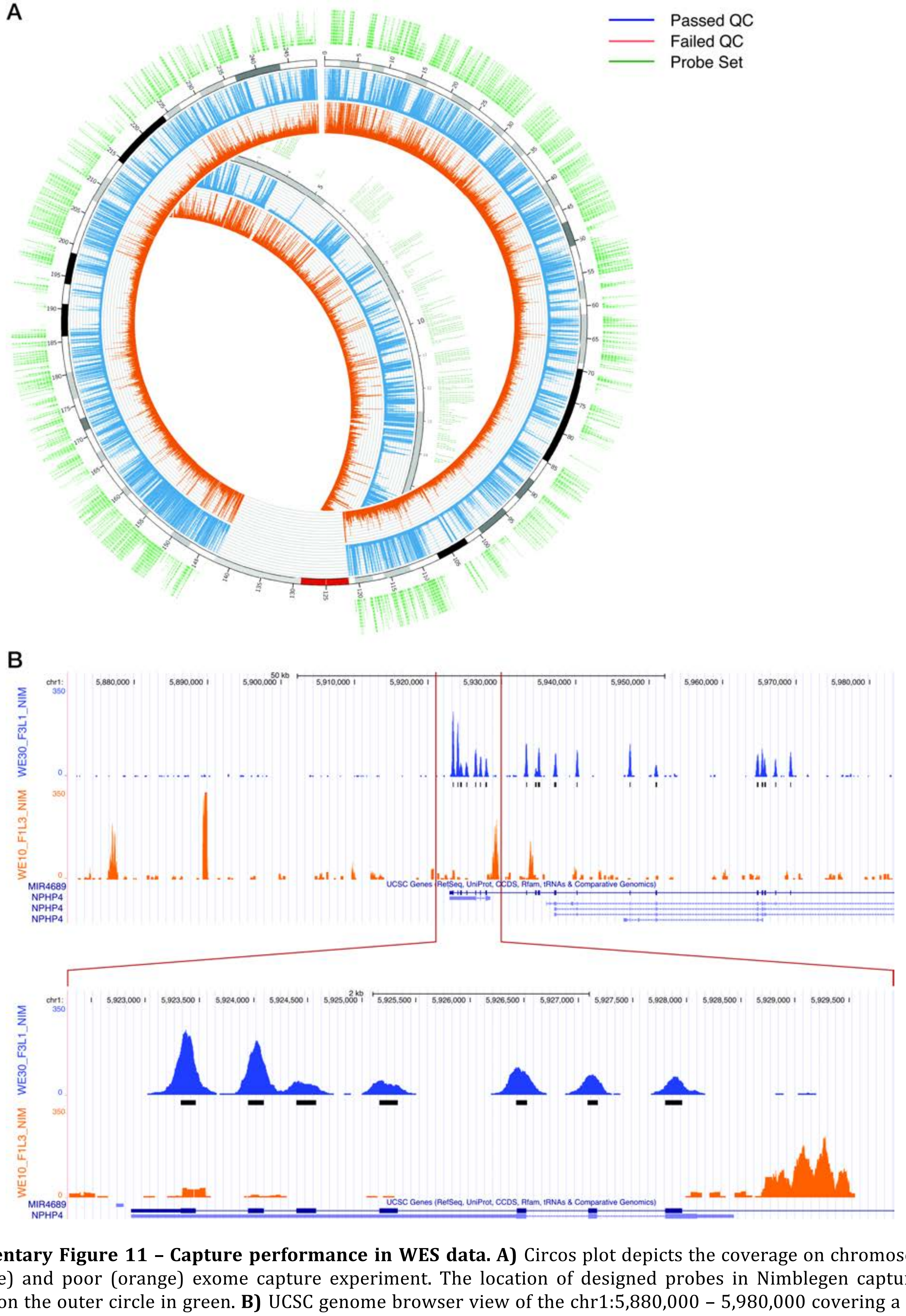

Supplementary Figure 11 – Capture performance in WES data. A) Circos plot depicts the coverage on chromosome 1 for good (blue) and poor (orange) exome capture experiment. The location of designed probes in Nimblegen capture kit are indicated on the outer circle in green. **B)** UCSC genome browser view of the chr1:5,880,000 – 5,980,000 covering a region on *NPHP4* gene for good and poorly captured whole exome sequencing data. Exons are indicated in black bars and the data coverage is illustrated in blue and orange for two WES data with good and poor capture, respectively.

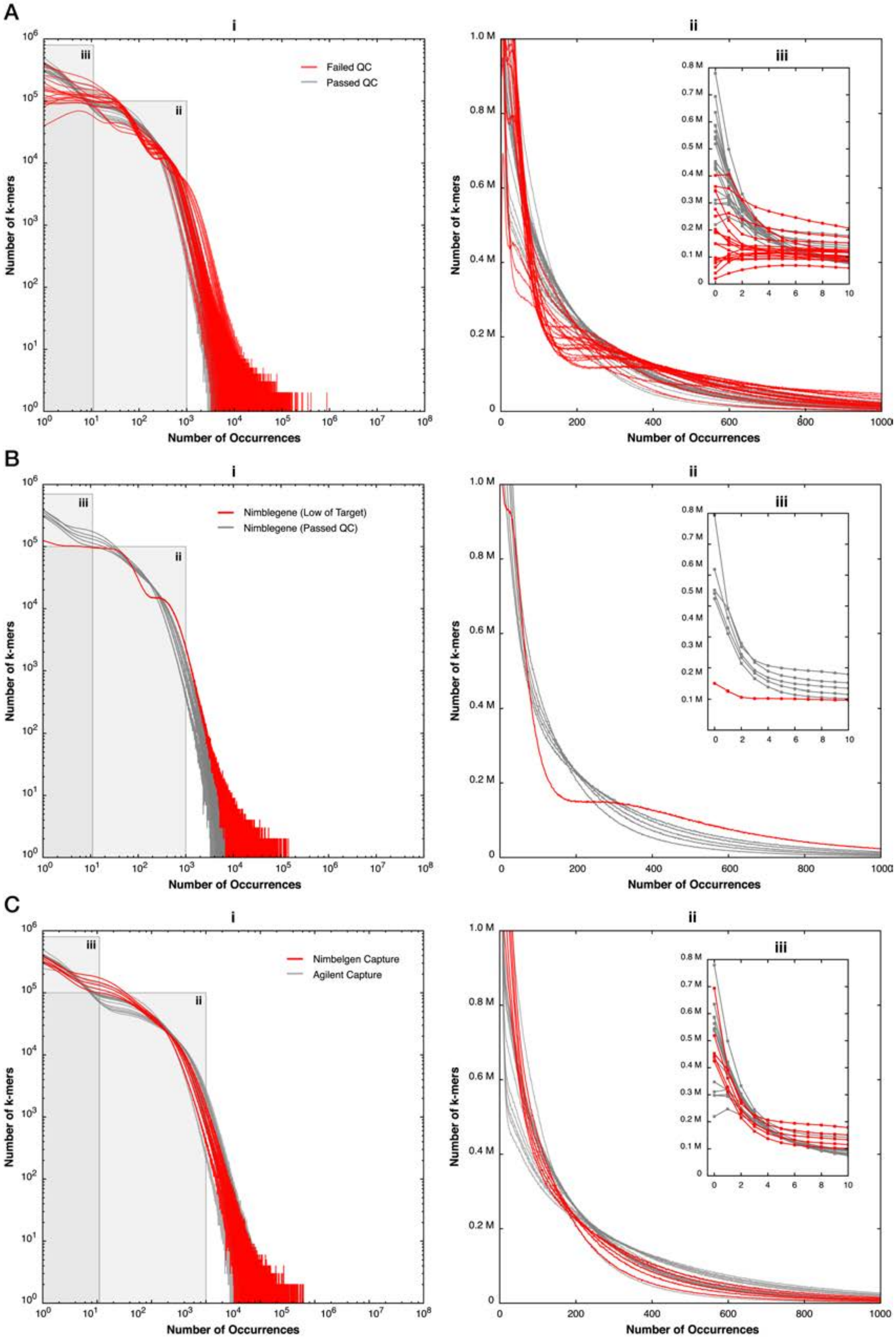

Supplementary Figure 12 – Data quality influences the complexity of the *k*-mer spectrum in WES data. A) The *k*-mer spectrum (k = 12) of samples with poor (red) or good (grey) capture performance. The full spectrum is depicted in panel **i** (left panel), and two zoomed in plots (**ii** and **iii**) are provided on the right. **B)** The *k*-mer spectrum of a failed sample along with successful captures using Nimblegen capture kit. **C)** The *k*-mer spectrum of samples that passed all QC measures grouped according to the choice of exome capture kit (Nimblegen in red and Agilent SureSelect in grey).

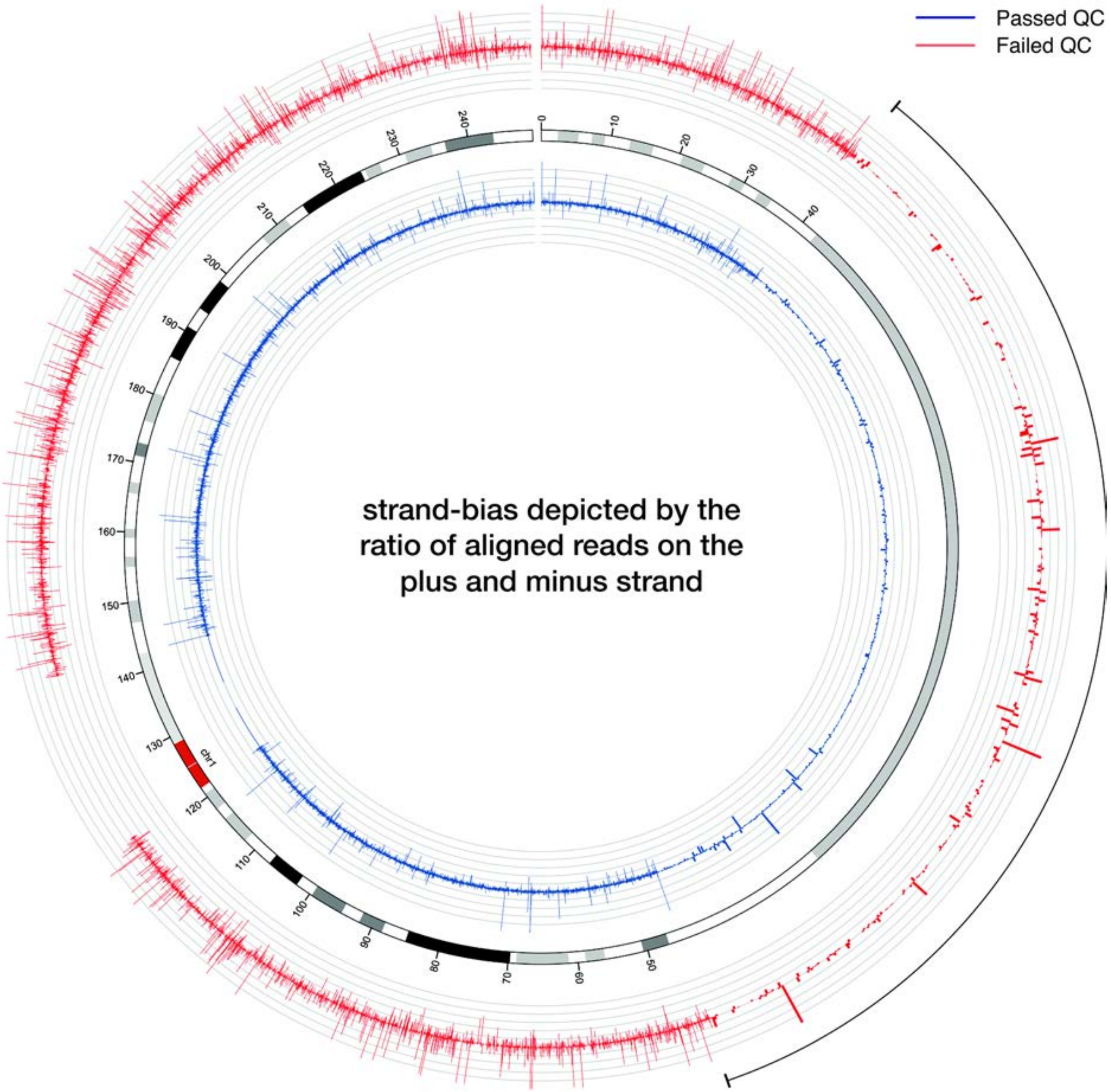

Supplementary Figure 13 – Strand-biased coverage in WES data is reflected in its *k*-mer spectrum. Circos plot illustrates the ratio between the number of reads that map to the plus or minus strand of the human reference genome. The data from WE10_F1L3_NIM with extreme duplication rate and subsequently imbalanced coverage of plus and minus strand is plotted on the outer circle in red. The result of another WES that passed all QC measures, with comparable number of reads, is plotted in the inner circle in blue. This plot illustrates the result on chromosome 1, partially zoomed in between coordinates 40 and 50 Mb.

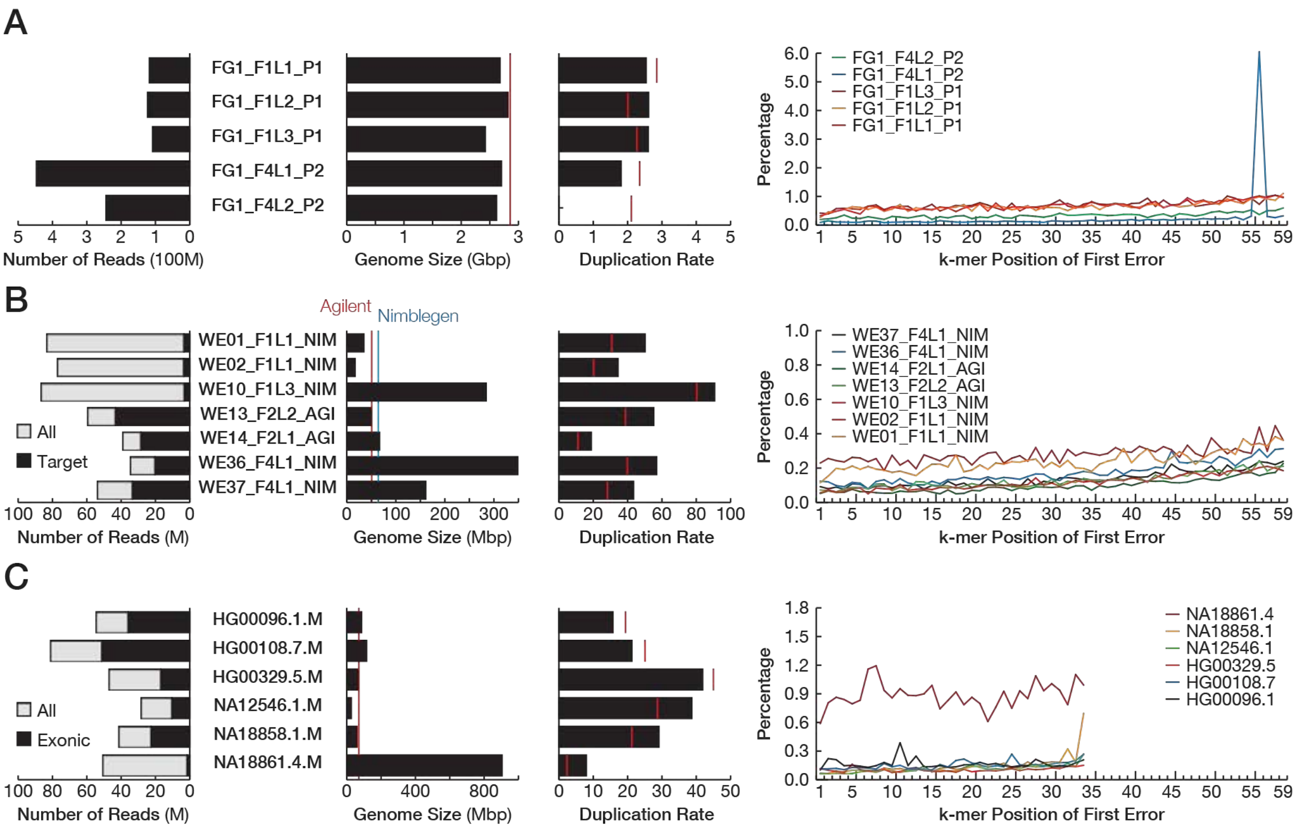

Supplementary Figure 14 – Detecting problematic samples using SGA. A) Total number of reads and estimated genome size and duplication rate of WGS data from the first individual using SGA. Red lines depict the expected genome size (excluding gaps) and duplication rate of each dataset that is calculated after alignment to the reference genome. Line plots show the frequency of the *k*-mer position of the first error for each dataset. Errors are estimated based on rarity of *k*-mer frequencies. **B)** Total number of reads and number of on-target reads are depicted for selected WES datasets along with SGA-estimated genome sizes and duplication rates. Red line depicts the targeted genomic region of the Agilent capture kit and the blue line depicts the targeted genomic region of the Nimblegen capture kit. Small red lines depict duplication rate of each dataset that is calculated after alignment to the reference genome. Line plots show the frequency of the *k*-mer position of the first error for each whole-exome dataset. **C)** Total number of reads and number of exonic reads in a set of RNA-Seq data along with SGA-estimated genome sizes and duplication rates. Red lines depict the total size of all exons in the human reference genome and duplication rate of each dataset that is calculated after alignment to the reference genome. Line plots show the frequency of the *k*-mer position of the first error for each of RNA-Seq datasets.

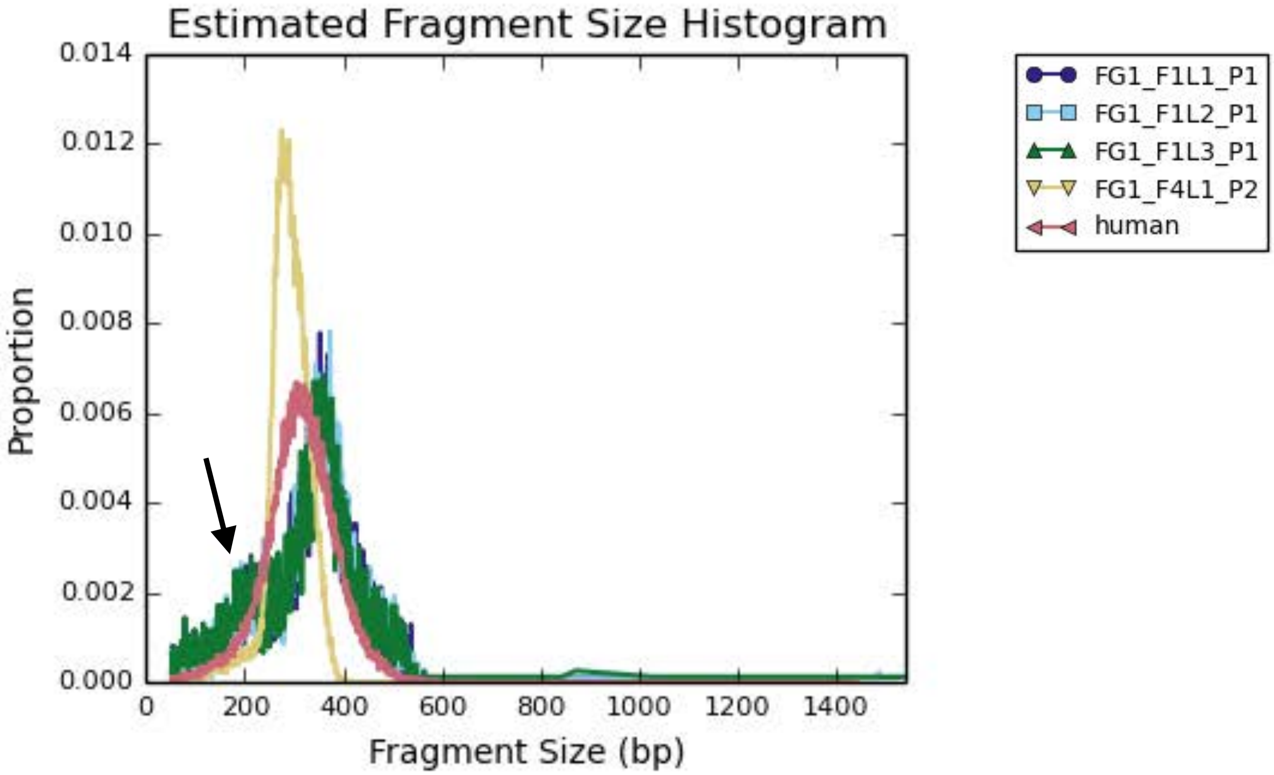

Supplementary Figure 15 – Estimated insert size distribution of WGS data using SGA. Fragment sizes are estimated by SGA-Preqc for a subset of WGS data. Green, light blue and dark blue lines depicted samples that are prepared using the first protocol (FG1_F1L1_P1, FG1_F1L2_P1 and FG1_F1L3_P1). Red and yellow lines depict samples that are prepared using the second protocol (FG1_F4L1_P2 and FG1_F4L2_P2). The arrow indicates the extra mode that makes the insert size distribution of samples from the first protocol bimodal.

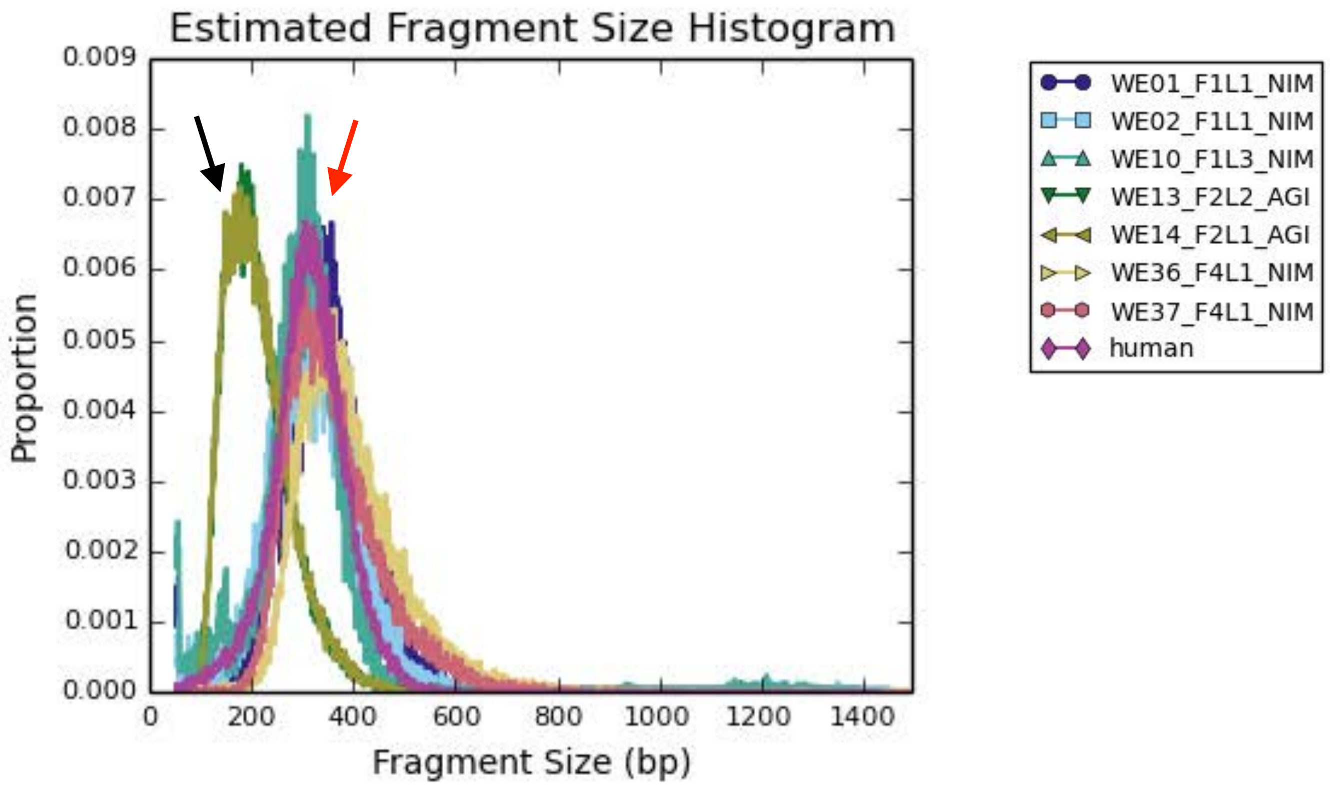

Supplementary Figure 16 – Estimated insert size distribution of WES data using SGA. Fragment sizes are estimated by SGA-Preqc for a subset of WES data. The black arrow indicates samples that are prepared using the Agilent SureSelect capture kit. The red arrow indicates samples that are prepared using the Nimblegen capture kit.

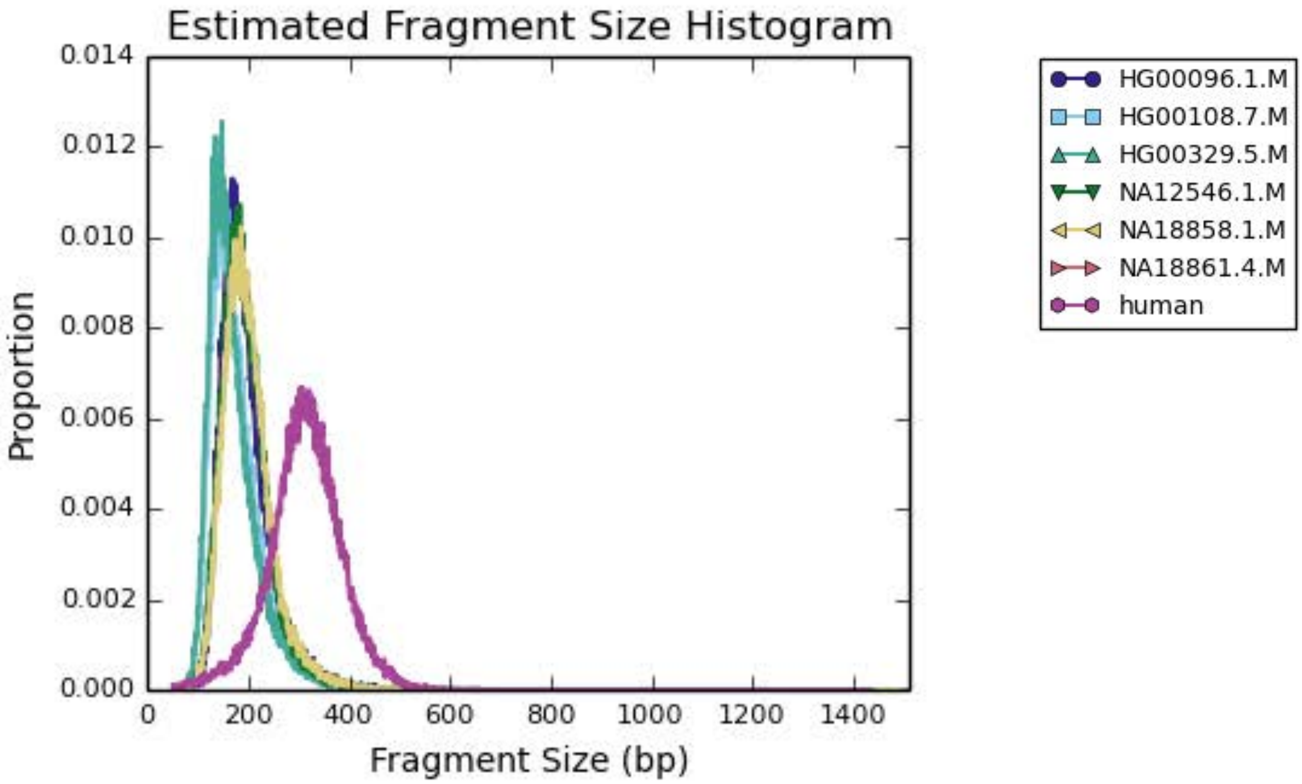

Supplementary Figure 17 – Estimated insert size distribution of RNA-Seq data using SGA. Fragment sizes are estimated by SGA-Preqc for a subset of RNA-Seq data. The WGS human data (purple), provided by SGA, is included as a reference.

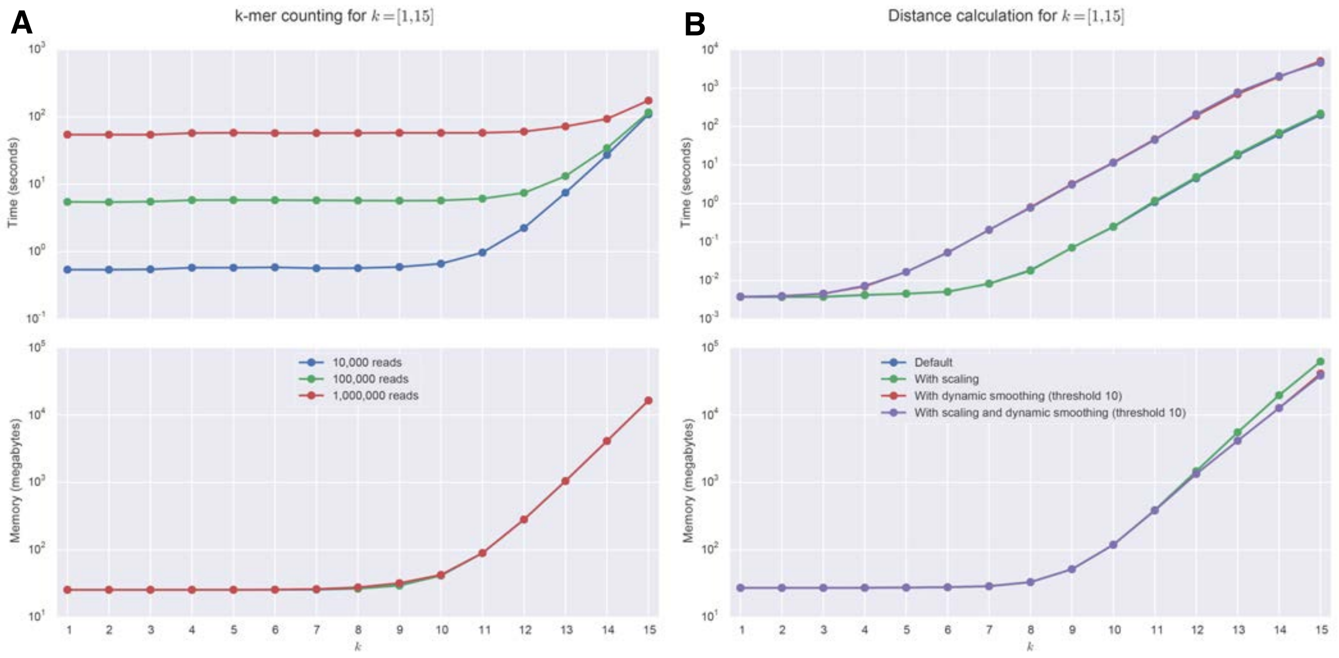

Supplementary Figure 18 – Speed and memory usage of kMer in generating and comparing profiles. A) Plots depict the time (second) and memory (MB) needed to generate *k*-mer profiles for various sizes (*k* = 1:15). **B)** Plots depict the time and memory needed to perform a pairwise comparison. During the pairwise comparisons, the process is repeated based on different use of scaling and smoothing functions. Datasets contain 10,000 to 1 million single-end reads of 100bp long. All tasks were carried out on a cluster node with Intel Xeon E5540 at 2.53 GHz with 8 cores, although only one core is used by kMer. For the most up-to-date and detailed documentation on performance and best practices visit http://kmer.readthedocs.org and www.lgtc.nl/kMer.

References

1. Chor, B., Horn, D., Goldman, N., Levy, Y. and Massingham, T. (2009) Genomic DNA k-mer spectra: models and modalities. *Genome biology*, **10**, R108.
2. Csuros, M., Noe, L. and Kucherov, G. (2007) Reconsidering the significance of genomic word frequencies. *Trends in genetics : TIG*, **23**, 543-546.
3. Hariharan, R., Simon, R., Pillai, M.R. and Taylor, T.D. (2013) Comparative analysis of DNA word abundances in four yeast genomes using a novel statistical background model. *PloS one*, **8**, e58038.
4. Herold, J., Kurtz, S. and Giegerich, R. (2008) Efficient computation of absent words in genomic sequences. *BMC bioinformatics*, **9**, 167.
5. Acquisti, C., Poste, G., Curtiss, D. and Kumar, S. (2007) Nullomers: really a matter of natural selection? *PloS one*, **2**, e1022.
6. Josse, J., Kaiser, A.D. and Kornberg, A. (1961) Enzymatic synthesis of deoxyribonucleic acid. VIII. Frequencies of nearest neighbor base sequences in deoxyribonucleic acid. *The Journal of biological chemistry*, **236**, 864-875.
7. Sved, J. and Bird, A. (1990) The expected equilibrium of the CpG dinucleotide in vertebrate genomes under a mutation model. *Proceedings of the National Academy of Sciences of the United States of America*, **87**, 4692-4696.
8. Subramanian, S. and Kumar, S. (2003) Neutral substitutions occur at a faster rate in exons than in noncoding DNA in primate genomes. *Genome Res*, **13**, 838-844.
9. Kosters, W.A. and Laros, J.F.J. (2008) In Bramer, M., Coenen, F. and Petridis, M. (eds.), *Research and Development in Intelligent Systems XXIV*. Springer London, pp. 293-303.
10. Lappalainen, T., Sammeth, M., Friedlander, M.R., t Hoen, P.A.C., Monlong, J., Rivas, M.A., Gonzalez-Porta, M., Kurbatova, N., Griebel, T., Ferreira, P.G. *et al.* (2013) Transcriptome and genome sequencing uncovers functional variation in humans. *Nature*, **advance online publication**.
11. t Hoen, P.A.C., Friedlander, M.R., Almlof, J., Sammeth, M., Pulyakhina, I., Anvar, S.Y., Laros, J.F.J., Buermans, H.P.J., Karlberg, O., Brannvall, M. *et al.* (2013) Reproducibility of high-throughput mRNA and small RNA sequencing across laboratories. *Nat Biotech*, **advance online publication**.
12. Caporaso, J.G., Lauber, C.L., Costello, E.K., Berg-Lyons, D., Gonzalez, A., Stombaugh, J., Knights, D., Gajer, P., Ravel, J., Fierer, N. *et al.* (2011) Moving pictures of the human microbiome. *Genome biology*, **12**, R50.
